# Polypharmacology of an Optimal Kinase Library

**DOI:** 10.64898/2026.03.17.711623

**Published:** 2026-03-19

**Authors:** Caitlin E. Mills, Clemens Hug, Karuna Anna Sajeevan, Nicholas Clark, Chiara Victor, Mirra Chung, Sameer Rawat, Bree Aldridge, Mark W. Albers, Ratul Chowdhury, Benjamin M. Gyori, Peter K. Sorger

**Affiliations:** 1 Laboratory of Systems Pharmacology, Harvard Program in Therapeutic Science, Harvard Medical School, Boston, MA, 02115, USA; 2 Department of Systems Biology, Harvard Medical School, 200 Longwood Avenue, Boston, MA 02115, USA; 3 Department of Chemical and Biological Engineering, Iowa State University, Ames, IA 50011, USA; 4 Current address: Department of Host-Microbe Interactions, St. Jude Children’s Research Hospital, Memphis, TN, USA; 5 Current address: Lyell Immunopharma, Inc., Seattle, WA, USA; 6 Department of Molecular Biology and Microbiology, Tufts University School of Medicine and Stuart B. Levy Center for Integrated Management of Antimicrobial Resistance, Boston, MA, USA; 7 Department of Biomedical Engineering, Tufts University School of Engineering, Medford, MA, USA; 8 Department of Neurology, Massachusetts General Hospital, Charlestown, MA, USA; 9 Khoury College of Computer Sciences, Northeastern University, Boston, MA, USA; 10 Department of Bioengineering, Northeastern University, Boston, MA, USA; 11 Ludwig Centre at Harvard, Harvard Medical School, Boston, MA, 02115, USA

## Abstract

Despite decades of research, current understanding of the spectrum of targets bound by kinase inhibitors remains incomplete. This complicates mechanism of action studies, drug repurposing, and understanding of adverse responses. Here, we describe kinome-wide profiling of an optimal kinase library (OKL) comprising 192 small molecules selected based on stage of clinical development, chemical diversity, and target coverage. Our results show that polypharmacology is widespread among kinase inhibitors independent of regulatory approval. The generally understood (“assigned”) targets of approved molecules are not necessarily the most potently inhibited and off targets include multiple understudied kinases. Moreover, median selectivity has not increased over time. We illustrate the use of synoptic OKL-kinome profiles in identifying potential toxicity targets, repurposing anti-inflammatory drugs for neurodegenerative and infectious diseases, and performing chemical genetic studies. Our studies illustrate how much remains to be discovered about the chemistry and biology of one of the largest classes of human therapeutics.

## INTRODUCTION

Optimizing the selective binding of a chemical compound to a desired protein target is a key step in small molecule drug development. This is particularly true in the case of targets that are members of multi-protein families. The human kinome comprises ~530 proteins (with the number somewhat dependent on definition)^1^ and as of 2026, over 100 kinase inhibitors have been FDA approved for use as therapeutics^2^ with an additional 369 compounds against 129 targets undergoing clinical trials^3,4^. This represents one of the most active areas of drug discovery: there were more kinase inhibitors approved by the FDA in 2025 than in any prior year^5^. In current practice, kinase inhibitors are most commonly designed to bind one or a small set of closely related members of the kinome (we refer to these as “assigned” targets). It is therefore common to describe drug ‘*A*’ as an inhibitor of assigned target ‘*B*’, but it is generally understood that binding to other targets (polypharmacology) plays an essential – if poorly understood – role in drug activity. Kinase inhibitors achieve regulatory approval based on clinical endpoints, survival in the case of anti-cancer drugs, but a description of their mechanism of action is an essential component of regulatory filings, and this invariably emphasizes the presumed role of assigned target(s). A complete understanding of how inhibition of other potential targets contributes to the clinical success of small molecules often occurs post-approval and is broadly dependent on better ways of assessing polypharmacology.

Both academic and commercial platforms exist to profile the binding affinities of small molecules against the great majority (~75%) of all protein kinases. Several high-impact projects have studied the polypharmacology of kinase inhibitors at scale^6–10^ using compound libraries that vary in composition. Despite a steady stream of drug approvals, it has been some time since kinase inhibitor polypharmacology has been studied in depth. This paper also exploits cheminformatics tools able to assemble libraries that strike a balance between chemical diversity, selectivity, stage of clinical development, and library size (and thus, cost). The resulting 192 compound Optimal Kinase Library (OKL) is one example of a library optimized on several competing characteristics.

The literature on protein kinase inhibitors is large, and affinity data obtained from the literature are actively curated in public databases such as ChEMBL^11^, IDG Pharos^12^ and BindingDB^13^. Metrics such as affinity spectra (TAS)^14^ consolidate available data on inhibitor binding and non-binding relationships in public data while accounting for uncertainty in the underlying evidence. However, systematic analysis of polypharmacology is not as simple as assembling literature knowledge. For kinase inhibitors, this literature relies on a wide range of assay types (e.g. enzymatic inhibition, affinity mass spectrometry, binding to recombinant protein libraries) and conditions (e.g. ATP or substrate concentration) with few overlapping data points. This makes kinome-wide analysis difficult. Moreover, affinity screening is often biased toward assigned target(s), suspected off-targets, and “toxicity” targets known to limit clinical utility. Data on “known non-binders” is particularly sparse, even though it is essential for establishing selectivity^14^. Thus, existing knowledge is too incomplete to answer basic questions about the breadth and significance of compound polypharmacology.

In this paper we use the OKL and Eurofins’ KINOMEscan platform^15^ to collect kinome-wide affinity data at four concentrations for 192 kinase inhibitors optimized for selectivity, affinity, and approval status. We introduce a Bayesian inference framework to estimate Kds for all inhibitor-kinase pairs, enabling inference of affinities outside tested concentrations. We find that OKL compounds have various degrees of polypharmacology and inhibit all assayed kinases (including pathogenic kinases from tuberculosis and malaria). Kinase clustering based on OKL inhibitor binding data differs from the familiar view of the kinome tree based on sequence similarity, demonstrating that sequence alignment is an imperfect way of thinking about polypharmacology. We illustrate the utility of the fully annotated OKL as a screening tool by using it to dissect, identify and characterize neuroprotective agents active in a model of Alzheimer’s disease, investigate vulnerabilities in a model of cisplatin-resistant ovarian cancer, and identify inhibitors of non-mammalian pathogenic kinases. These use cases show that systematic kinome-scale knowledge of kinase polypharmacology has broad utility across biological contexts.

## RESULTS

### Establishment of kinome-wide affinities for a practically scaled Optimal Kinase Library

**Figure 1:**
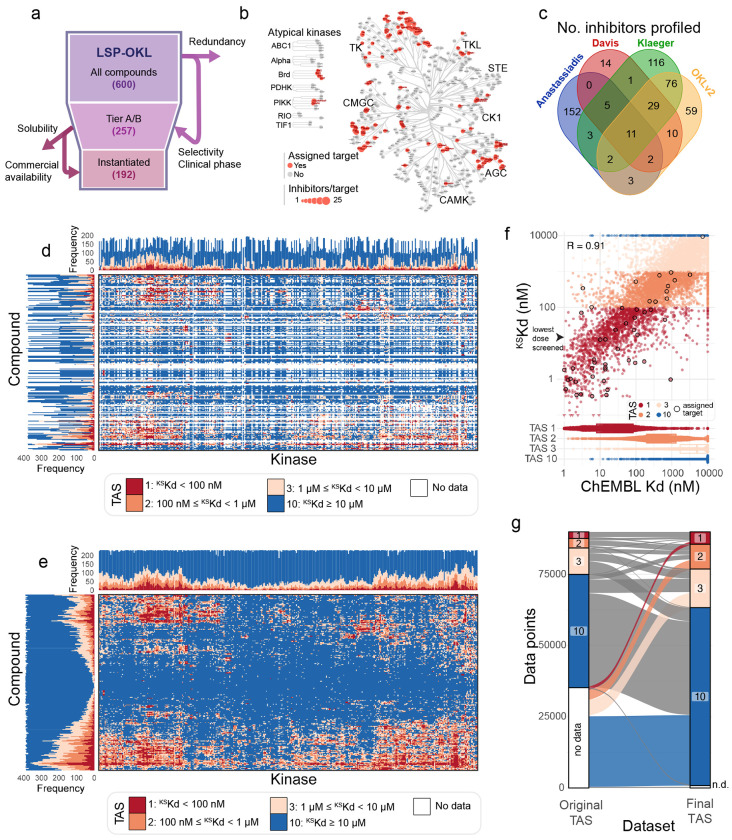
A complete view of kinome-wide affinities for the 192 compound ‘Optimal Kinase Library’. **(a)** Schematic representation of the reduction of OKL from 600 compounds to the instantiated OKL set of 192 compounds. **(b)** The nominal targets of OKL compounds shown on the kinome tree; red indicates a nominal target and the size of the spot indicates the number of inhibitors per target. **(c)** Venn diagram showing the overlap between the inhibitors profiled in similar efforts and those in OKL. **(d)** Kinome-wide target affinity spectra based on ChEMBL data for all OKL compounds prior to the current study. **(e)** Kinome-wide target affinity spectra for all OKLv2 compounds after the addition of the data generated in the current study. **(f)** Scatterplot comparing the ^KS^Kd values measured in this paper to those previously available in ChEMBL. Data points are colored by TAS value, and the values for each compound’s assigned target(s) are outlined in black. **(g)** An alluvial plot depicting the knowledge gained from addition of the data generated in the current study. TAS values on the left are from (c) and on the right from (d).

The larger and more diverse a chemical library and the more comprehensive the data on activity the greater the insight into compound-target relationships. However, collecting kinome-wide affinity data is expensive; for 10-point enzymatic assays this is ~$100/curve or $10M for 200 compounds against 500 targets. The KINOMEscan^®^ kinome-scale profiling assay available from Eurofins is 5-10-fold cheaper and has therefore been used in more than 2,000 publications. Systematic profiling is nonetheless sufficiently expensive to require compact libraries^6–9,14,16–18^. Thus, the OKL in this work aimed for a minimal collection balancing target coverage and structural diversity so that, ideally, each kinase was bound by two inhibitors with the highest possible selectivity and the greatest difference in chemical structure (i.e. Tanimoto similarity <= 0.2 between pairs based on Morgan fingerprints with 2 Å radius)^14^. When a choice was available, we selected compounds that were approved for use in humans. This resulted in a 192-compound library comprising 44 FDA-approved drugs, 98 molecules that have been, or are currently, in clinical trials, and 50 pre-clinical “tool” compounds having a total of 115 unique assigned targets (Figure 1a-b). Overlap between libraries previously subjected to systematic kinome profiling and OKL was surprisingly limited (Figure 1c) due to differences in study goals, our emphasis on target and chemical diversity, and a steady increase in the scope of clinical approvals^6–9,17^. For this reason, and those outlined above, knowledge of kinome-wide binding by OKL inhibitors was sparse (Figure 1d).

KINOMEscan scanMAX profiling (hereafter KINOMEscan) is based on a competitive binding assay comprising 406 wildtype (WT) human kinases and 59 kinases carrying oncogenic or resistance mutations^19,20^ plus three non-mammalian kinases (the availability of kinases is dependent in part on the feasibility of making them recombinantly). In KINOMEscan, a compound of interest is incubated with oligo-tagged recombinant kinases and immobilized ATP-like ligands capable of binding all kinases in the library; the amount of ligand-bound kinase is measured by qPCR to generate a ‘percent of control’ value (relative to incubation with DMSO); a value of 0 denotes strong binding and 100 no binding. We assayed each OKL inhibitor at four concentrations spanning four orders of magnitude (10 μM, 1 μM, 100 nM and 12.5 nM) to generate 89,856 four-point dose response curves (192 OKL compounds x 468 targets). Quality control (see Methods, Supplementary Figure 1) revealed ~900 *discordant* dose response profiles (~1% of the data) in which percent control did not fall with increasing inhibitor concentration (Supplementary Figure 1). These data were omitted from further analysis.

**Figure 2:**
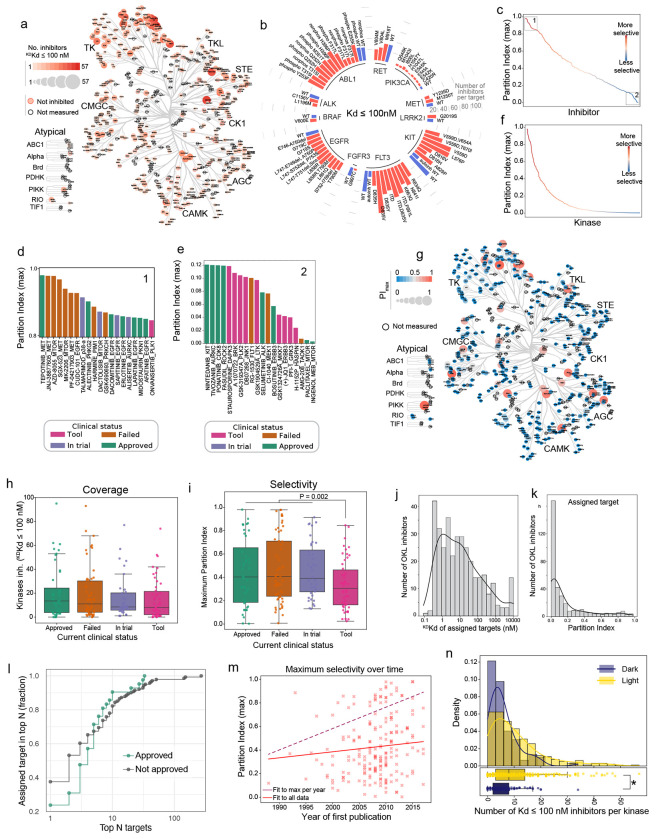
Kinome coverage and selectivity of OKL inhibitors. **(a)** The number of OKL inhibitors (^KS^Kd < 100 nM) per kinase shown on the kinome tree by the size and intensity of the markers. Kinases not inhibited are outlined in red, kinases not assayed are outlined in black. **(b)** Radial bar plot showing the number of OKL inhibitors with ^KS^Kd ≤ 100 nM for all mutant kinases (red bars) assayed in the KINOMEscan panel. WT kinases are indicated with blue bars. **(c)** The maximum partition index (PI_max_) for each inhibitor in OKL. The inset barplots show the most (top) and least (bottom) selective inhibitors and associated kinase target colored by current clinical status. **(d)** PI_max_ for each kinase. The inset CORAL tree shows the PI_max_ for each kinase, the darker and redder the circle, the more selectively that kinase is inhibited by an OKL compound. **(e)** Boxplot showing the number of kinases per inhibitor with ^KS^Kd ≤ 100 nM by clinical status. **(f)** Boxplot showing the maximum partition index for each OKL inhibitor by clinical status. P-value is from a Welch’s t-test comparing tool compounds to all others. **(g)** Histogram of the ^KS^Kd values for the assigned target(s) of OKL compounds. **(h)** Histogram of the partition indices for the assigned target(s) of OKL compounds. **(i)** Cumulative stairstep plot showing how often the nominal target is in the top N targets by ^KS^Kd for approved (green) and not approved (gray) inhibitors. **(j)** Scatterplot showing PI_max_ with respect to the year each compound was first published. The lines of best fit to all data points and to the highest datapoint per year are shown in solid red and dashed maroon, respectively. **(k)** Histogram showing the number of OKL inhibitors that bind each dark and illuminated kinase with ^KS^Kd ≤ 100 nM. Boxplots representing the same data are shown below the histograms. The * indicates Welch’s t-test P < 0.0001.

We explored a variety of approaches for estimating KINOMEscan-derived dissociation constants (^KS^Kd) and settled on Bayesian inference. Bayesian modeling substantially improved the reliability of (^KS^Kd) estimations compared to linear interpolation, particularly when values were below the lowest concentration tested experimentally (12.5 nM; see Supplementary Note) and resulted in a more complete view of kinome-wide binding for OKL inhibitors (Figure 1e). Those inhibitor-kinase pairs for which data were available in ChEMBL exhibited good agreement (R= 0.91) with ^KS^Kd estimates (Figure 1f-g). Two key features of a library of small molecule inhibitors are coverage – how many targets are bound by how many inhibitors; and selectivity – how many targets are preferentially (selectively) bound by any inhibitor. OKL achieve full coverage of all 468 assayed targets at a relatively high inhibitor concentration of 1 μM or below (2-130 inhibitors per kinase at ^KS^Kd ≤ 1 μM) and 94% coverage of WT kinases at 100 nM or below (0-57 inhibitors for 379/406 WT kinases at ^KS^Kd ≤ 100 nM); at this lower concentration all mutant kinases were also inhibited, likely because they have been the focus of years of intensive medicinal chemistry (Figure 2a-b, Supplementary Figure 2a–c). Note that these estimates of kinome coverage are substantially greater than what can be inferred from Eurofins’ standard recommendation that a binder is simply a molecule that achieves <35% of control activity at a single dose.

### Kinase inhibitor selectivity for tool compounds and human therapeutics

Several metrics are available for quantifying library selectivity;^6,21,22^ we used the easily-understood partition index (PI), which represents the fraction of an inhibitor bound to each kinase (having a ^KS^Kd estimate) in a theoretical situation in which all kinases are present in excess^21^. A perfectly selective inhibitor binding a single target would have a maximum PI (PI_max_) = 1 and an inhibitor binding many targets with similar affinity would have PI_max_ << 1. As an example, PI_max_ values for OKL inhibitors spanned a range from < 0.1 for AMG-208 to > 0.98 (for tepotinib binding to the MET receptor tyrosine kinase; RTK) (Figure 2c-e). We identified 69 “highly selective” compounds that bound to 37 WT kinases with PI_max_ > 0.5, but maximum selectivity for most kinases was PI_max_ < 0.2 (Figure 2f-g). This suggests that they are only bound by compounds that inhibit other kinases at similar concentrations.

Using OKL-KINOMEscan data, we examined four prevailing assumptions about the development of kinase inhibitors as human therapeutics: (i) approved drugs are more selective than compounds that failed in clinical trials (where ‘failed’ is defined as a trial compound not approved and no longer under investigation^3^); (ii) assigned targets are generally potently and selectively inhibited, particularly for FDA-approved drugs; (iii) compound selectivity has increased over time; and (iv) understudied (dark) kinases (per the definition of the NIH Investigating the Druggable Genome project)^23^ are less widely inhibited than well-characterized (illuminated) kinases.

First, we grouped compounds based on maximum reported clinical trial phase, approval status, or trials registered in ClinicalTrials.gov and found that FDA-approved drugs do not differ from compounds in other stages of clinical development in terms of coverage or selectivity; however, purely pre-clinical tool compounds are on average less selective (Figure 2h-i, Supplementary Figure 2d–e). Second, while most assigned targets are bound with high affinity (Figure 2g, ^KS^Kd ≤ 10 nM for 48% of assigned targets), selectivity varied widely (PI = 0 to 0.98, median 0.06, mean 0.17; Figure 2k) suggesting that many compounds are potent but not highly selective. Considering only FDA-approved drugs, an assigned target was the *highest* affinity target in only 23% of cases examined; for compounds that are not FDA-approved the proportion was 37% (Figure 2l). Thus, if we define a promiscuous inhibitor as one that that binds many kinases, and a promiscuous kinase as one that binds many inhibitors, profiling of the OKL library revealed a wide range of promiscuity in inhibitors and kinases, even among FDA-approved drugs. Moreover, if we use the year of first publication to estimate the “age” of an OKL compound we find that average selectivity has not changed significantly over time. Thus, high selectivity (PI_max_ value) has not been necessary for past clinical success with kinase inhibitors. However, the highest selectivity achieved by a single compound has increased (Figure 2m; dark dotted line) reflecting the presence of outliers in the data presumably arising from advances in kinase inhibitor medicinal chemistry.

### Kinase anti-targets and dark kinases

The concept of an anti-target, a protein that must be spared during drug discovery, is exemplified by the cardiac K+ channel hERG^24^. It has been proposed that some kinases also represent anti-targets^25,26^. Although no definitive list of kinase anti-targets has as-yet been assembled, unwanted inhibitor interactions should be identifiable in OKL-KINOMEscan data. One striking and unreported example involves alectinib (approved as Alecensa^®^ for metastatic non-small cell lung cancer) which has ALK as an assigned target. OKL-KINOMEscan suggests that alectinib can also bind PHKG2, the liver-specific γ-subunit of the phosphorylase kinase; using enzymatic assays we confirmed that alectinib is an IC50= 33 nM inhibitor of PHKG2 (Supplementary Figure 2h). Mutation in PHKG2 give rise to severe liver disease^27^ and inhibition of the kinase by sunitinib causes liver toxicity^28^. Hepatotoxicity leading is a recognized problem in up to 60% of patients treated with alectinib^29^ (with grade 2-4 toxicity in 5-8% of patients) and we speculate that this is a consequence of inhibition of PHKG2. Examination of ^KS^Kd values for ALK inhibitors across the OKL-KINOMEscan dataset show that a subset also target PHKG2 but that others do not. It should therefore be possible to create potent ALK inhibitors in which PHKG2 is not bound, thereby reducing hepatoxicity.

Wide differences in the levels of attention given to kinases over time has given rise to the concept of “understudied” or dark kinases^30^ (the focus of a major NIH program)^31^. We found that these understudied kinases^23^ were widely bound and selectivity inhibited even by FDA-approved compounds (Figure 2n, Supplementary Figure 2f, g). The number of OKL compounds inhibiting dark kinases was less than for illuminated kinases however (median of 5 vs 8 inhibitors per kinase at a threshold of ^KS^Kd ≤ 100 nM, respectively). These largely uncharacterized inhibitory activities represent a potential starting point for targeting understudied kinases and may also provide insight into efficacious off-target activities of existing human therapeutics.

### Kinase promiscuity is associated with a DFG-out conformation binding Type I and II inhibitors

**Figure 3:**
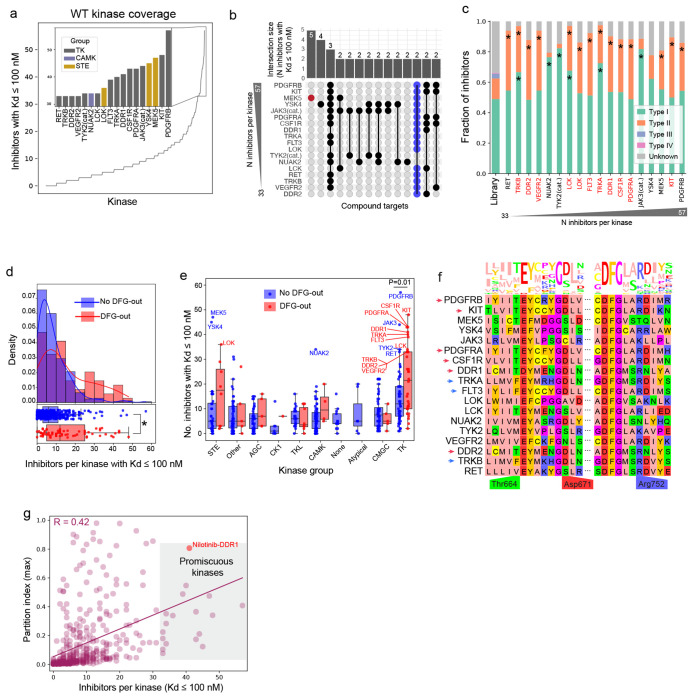
A subset of kinases are promiscuous for OKL inhibitors. **(a)** The number of OKL inhibitors (^KS^Kd ≤ 100 nM) per kinase. The top 15 ranks with ties are shown in the inset bar plot colored by kinase group. These 18 kinases are referred to as promiscuous. **(b)** Upset plot showing the number of overlapping inhibitors with ^KS^Kd ≤ 100 nM for the promiscuous kinases. **(c)** Stacked bar graph showing the distribution of inhibitor types in OKL and that bind each promiscuous kinase. Kinases identified as promiscuous in a previous study are in red. **(d)** Histograms of the number of inhibitors per kinase with ^KS^Kd ≤ 100 nM for kinases with (red) and without (blue) reported DFG-out structures. KDE plots are overlaid for visualization. Boxplots representing the same data are shown below the histograms. The * indicates Welch’s t-test P < 0.0001. **(e)** Boxplots of the number of inhibitors with ^KS^Kd ≤ 100 nM for kinases with (red) and without (blue) DFG-out structured by kinase group. Welch’s t-tests were performed on all pairs, P-values are only shown where P < 0.05. **(f)** Multiple sequence alignment and sequence logo plot of the promiscuous kinases. Residues previously implicated in RTK promiscuity are labeled. Amino acids are colored by the zappo color scheme. **(g)** Scatter plot of the number of inhibitors per kinase with ^KS^Kd ≤ 100 nM with respect to the kinase’s maximum partition index. Promiscuous kinases are in the shaded gray box.

What is the basis of promiscuous binding of some human kinases to ATP-competitive inhibitors? To address this, we focused on the 18 most promiscuous kinases in our dataset (15 TKs, two STE kinases, and one CAM kinase; Figure 3a) each of which bound ≥ 33 compounds with relatively high affinity (^KS^Kd ≤ 100 nM). Overlap among these inhibitors was limited with at most two inhibitors binding to 16/18 promiscuous kinases (Figure 3b, blue points); for example, MEK5 (Figure 3b, red point) was bound by five inhibitors that did not bind any other promiscuous kinases (at ^KS^Kd ≤ 100 nM). Thus, promiscous kinases do not simply bind a common set of promiscuous inhibitors.

Identified structural determinants of binding specificity for ATP-competitive kinase inhibitors focus on the conformation and local environment of a conserved three amino acid motif (DFG) that lies immediately N-terminal to the activation loop^32^. Kinases assume both a *DFG-in* conformation, which is generally catalytically active, and a *DFG-out* conformation, which is inactive. Kinase inhibitors are classified into type I inhibitors that bind both DFG-in and DFG-out conformations and type II inhibitors that bind only DFG-out conformations; type III and IV inhibitors (so-called “allosteric inhibitors”) bind outside the active site and are substantially less common^33^. We found that 14/18 promiscuous kinases were bound by more type II inhibitors than expected by chance (given OKL composition) (Figure 3c) consistent with binding to a DFG-out conformation. Moreover, kinases with a DFG-out structure in the Protein Data Bank (PDB)^34^ were more promiscuous than those without (Figure 3d, mean number of ^KD^Kd ≤ 100 nM inhibitors 15.5 and 8.3, respectively; Welch’s t-test P = 2.66 x 10^−5^). This difference was greatest for kinases in the STE and TK families (Figure 3e).

Six RTKs promiscuous for OKL compounds (PDGFRA, PDGFRB, KIT, CSF1R, DDR1, DDR2) were previously shown to bind many members of the 645-compound PKIS2 library, which has no overlap with OKL^35,16^. This was attributed to stabilization of the DFG-out conformation by a D671 to R752 salt bridge (the numbering is based on the DDR1 kinase)^35^ (Figure 3f, red arrows). In our study, three additional RTKs (TRKA, TRKB and FLT3) having analogous D671 and R752 residues (Figure 3f, blue arrows) were also promiscuous. However, the same residue pair is present in four kinases that are not RTKs and are not promiscuous suggesting the impact of this feature on inhibitor binding is limited to RTKs. Thus, kinase promiscuity is associated with binding to the DFG-out conformation and for RTKs this is stabilized by a D671 to R752 salt bridge. Since several of these proteins are being targeted for development of human therapeutics, we asked whether promiscuity is incompatible with selective inhibition. Our data suggest that the answer is no: DDR1, for example, is bound by 41 OKL compounds (^KS^Kd ≤ 100 nM) but one of these (Nilotinib, whose assigned target is BCR-ABL) is highly selective, with a partition index of 0.81 (Figure 3g, red point).

### Sequence homology and inhibitor binding provide distinct views of the kinome

**Figure 4:**
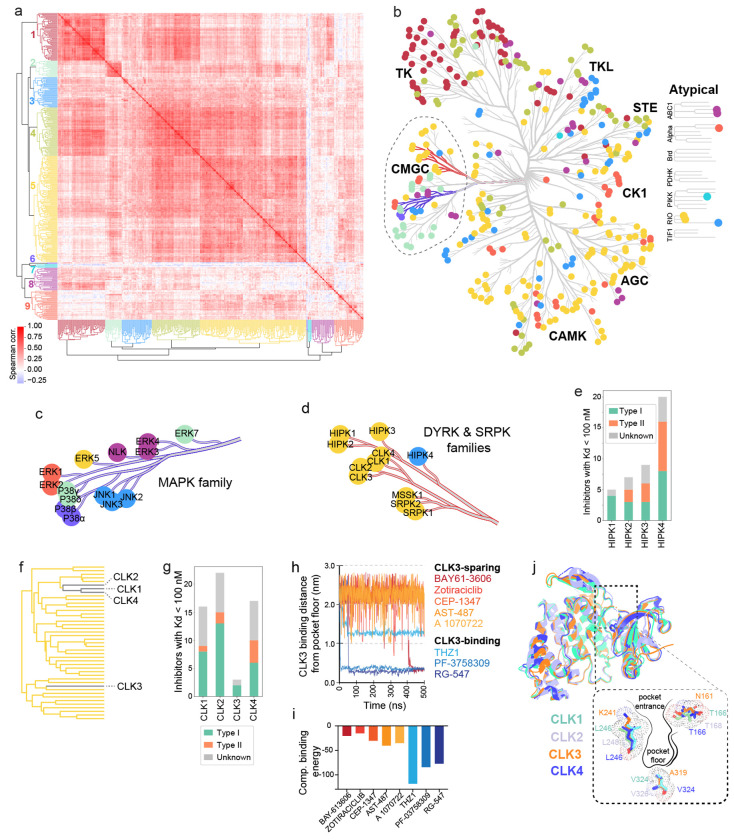
Kinase sequence homology with respect to OKLv2 compound affinities. **(a)** Hierarchical clustermap of the spearman correlation between kinases based on their ^KS^Kd values for all OKL compounds. **(b)** The colors of the clusters are overlaid on the kinome tree. The blue branches are magnified in **(c)** and the red branches in **(d)**. **(e)** Bar plot of the number of OKL inhibitors with ^KS^Kd ≤ 100 nM for each HIPK colored by inhibitor type. **(f)** Subset of the hierarchical tree from (a) showing the positions of each CLK. **(g)** Bar plot of the number of OKL inhibitors with ^KS^Kd ≤ 100 nM for each CLK colored by inhibitor type. **(h)** 500ns molecular dynamics trajectories showing the distance of the indicated inhibitors from the floor of CLK3’s binding pocket. **(i)** Computational binding energy scores from GROMACS molecular dynamics simulations of eight inhibitors with CLK3. **(j)** A visual overlay of the overall structures of CLK1,2,3, and 4 and zoomed-in view of their binding pockets highlighting key amino acids.

The classic kinome tree is based on classification by Manning, et al.^36^ in which kinases are clustered based on sequence homology (primarily in the catalytic domain). The tree is split into nine major groups (e.g. TKs, CAM kinases, AGC kinases, etc.) that each contain multiple families. Targeting specific isoforms within these families is a common goal of drug discovery, as exemplified by the search for PI3K inhibitors that discriminate between oncogenic kinases and immune regulators^37^. To see if OKL affinity data could assist in similar tasks, we performed hierarchical clustering based on the Spearman correlation of ^KS^Kd values. This yielded nine major clusters (Figure 4a, b) that differed substantially from the groups generated from sequence similarity (Figure 4b). Taking the CMGC group as an example (Figure 4b, dotted outline), the 14 kinases in the MAPK family form one sequencebased cluster but six OKL clusters (Figure 4b, blue branch; Figure 4c). Thus, proteins most related in sequence can have very different inhibitor binding profiles: ERK1 and ERK2 are bound by different OKL inhibitors than ERK3 and ERK4, for example. In contrast, a second branch in the CMGC group comprising DYRK and SRPK family kinases lie in a single OKL cluster with a single outlier: HIPK4 (Figure 4b, red branch; Figure 4d). A HIPK4 inhibitor (Figure 4e) is more likely to bind other kinases in OKL cluster 3 which includes c-Jun N-terminal kinases (JNK1, JNK2, JNK3) which are in the MAPK family (Figure 4a-d). Inhibitor selectivity therefore represents a different way of viewing the kinome tree than sequence similarity and can be used to prioritize off-targets for deeper analysis. With additional data, it should be possible to extend this idea and construct a kinome tree based entirely on small molecule binding as opposed to sequence similarity.

The CDC2-like kinases (CLKs) have been linked to diseases ranging from cancer to neurodegeneration,^38^ and CLK inhibitors are under investigation for multiple types of cancer. CLK1, CLK2, and CLK4 cluster tightly together based on OKL binding profiles but CLK3 - like HIPK4 - is an outlier relative to its closest homologues (Figure 4f, g). To investigate residues involved in small molecule binding by CLK3, we performed 500 ns all-atom molecular dynamics (MD) simulations for eight inhibitors having a range of affinities for CLK kinases. Starting the simulation with compounds outside the binding pocket, we found that high affinity CLK3 binders entered and remained in the pocket as measured by the distance from residue A319 on the pocket floor (< 0.4 nm for PF-3758309 and RG-547, and ~1.2 nm for THZ1; Figure 4h) and this corresponded to computational binding scores < −75 (Figure 4i). In contrast, inhibitors with ^KD^Kd ≥ 1000 nM for CLK3 remained distant (~2.5 nm) from the pocket floor over the 500 ns simulation suggesting that they failed to enter the pocket, thereby reproducing the experimental data. Simulations suggested that two residues differing between CLK3 and other CLKs at the pocket entrance are likely involved in this selectivity: T166→N161, L246→K241 (number based on CLK3 residue positions; Figure 4j). These residue positions are known, along with V324→A319 on the pocket floor, to affect the charge distribution and size of the drug/ATP binding pocket.^39,40^ Thus, subtle structural differences in kinase binding pockets that are not discernable by sequence alignment can be detected by OKL kinome profiling and potentially used to guide isoform-selective inhibitor development.

### Using an optimized kinase library and affinity data to explore nuerodegeneration

**Figure 5:**
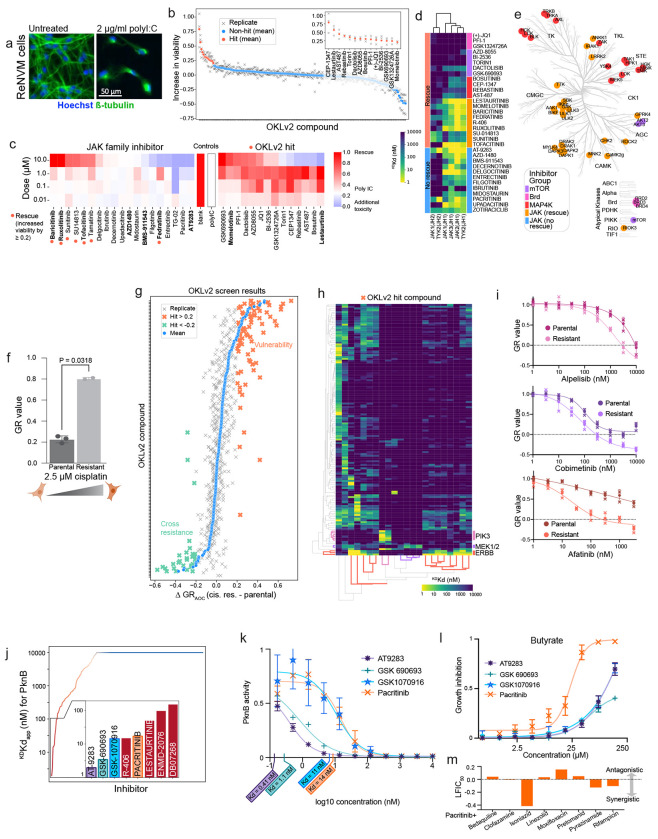
OKLv2 use cases. **(a)** Images of ReNcell^®^ VM cells under control conditions (left) and treated with 2 μg/ml polyI:C (right) for seven days. Nuclei are shown in blue and ß-tubulin in green. The scale bar is 50 μm. **(b)** The change in percent viability conferred by the addition of each OKLv2 compound at 1 μM to treatment with 2 μg/ml polyI:C relative to polyI:C alone. The means of biological triplicates (denoted with x’s) are shown as solid o’s in red for hits, and blue for all others. **(c)** Heatmap showing percent viability of ReNcell^®^ VM cells treated with polyI:C and increasing concentrations of JAK inhibitors, and the OKL hits identified in **(b)**. **(d)** ^KS^Kd values for the JAK family kinases for all inhibitors shown in **(c)** arranged by phenotype and common targets. **(e)** Common targets of rescuing agents shown on the kinome tree colored by the groups shown in **(d)**. **(f)** Bar graph showing GR values for parental and cisplatin resistant SNU8 cells treated with 2.5 μM cisplatin for 72 h. **(g)** Difference in GRAOC between cisplatin resistant and parental SNU8 cells for each OKL inhibitor (n=3 biological replicates). Higher GRAOC indicates sensitivity, while lower GRAOC indicates resistance. Individual replicates are shown with x’s, and the means with o’s. Hits are marked in bold orange if more effective and in bold green if less effective in the resistant cells. **(h)** Clustermap of ^KS^Kd values for hits that were more effective in resistant cells in at least two primary screens. Only kinases that were inhibited with ^KS^Kd ≤ 100 nM by at least one hit are shown. **(i)** Dose response curves for inhibitors of targets identified in **(h)** for parental and cisplatin-resistant SNU8 cells. **(j)** ^KS^Kd values for OKL compounds against PknB, the strongest inhibitors are shown in the inset bar graph. **(k)** 11-point Kd curves and Kd values for four of the top inhibitors from **(l)** for PknB. **(m)** Dose-dependent growth inhibition of Mtb treated with the same drugs in the butyrate growth condition. **(n)** Effect of pacritinib in combination with the drugs indicated on Mtb growth.

To explore practical uses of OKL data, we performed cell-based screening in three distinct biological contexts: (i) rescue of neuronal cell death in a model of Alzheimer’s disease (ii) identifying targets for killing of platinum resistant ovarian cancer cells and (iii) identification of inhibitors of non-mammalian kinases. A subset of AD is thought to involve neuronal death triggered by an inflammatory response to cytoplasmic double stranded RNA (cdsRNA).^41^ This can be recapitulated in a cell-based screen by treating differentiated ReNVM neurons with the dsRNA mimetic, polyI:C, which leads to type I interferon signaling and cell death. Neuronal death can be rescued to variable degrees by Janus family kinase inhibitors (the JAK family comprises JAK1-3 and TYK2)^41,42^ (Figure 5a). To determine if off targets were involved in rescue, we extended the OKL dataset by performing KINOMEscan profiling on 12 additional JAK inhibitors, focusing on the two most informative concentrations: 100 nM and 10 μM; the resulting OKL+JAKi dataset included a total of 21 inhibitors with assigned targets in the JAK family. We then screened the OKL at 1 μM in ReNVM cells treated with polyI:C and identified 14 inhibitors that conferred a significant (20% or greater) increase in viability (Figure 5b). In the subsequent rescreen, these 14 hits, and all compounds with one or more assigned targets in the JAK family (33 compounds in total), were then tested for rescue at four doses (10, 1, 0.1, 0.01 μM) (Figure 5c).

This yielded 21 rescuing molecules including 9/21 inhibitors whose assigned targets were JAK family kinases. Variability in rescue did not correlate with affinity for assigned JAK targets, which was generally in the nanomolar range (Figure 5c). The additional OKL hits did not detectably bind JAK family members (Figure 5d). We found that the rescuing agents, but not the non-rescuing agents, had micromolar ^KS^Kds for multiple kinases other than JAK1-3/TYK2 including, ULK1-3, the numb associated kinases GAK, AAK1 and BIKE, and the entire DAPK family DAPK1-3 and DRAK1-2 that function in endocytosis, viral trafficking, and autophagy (Figure 5e, Supplementary Figure 4c)^43,44^. This is consistent with recent data showing that numb associated kinases contribute to the activity of baricitinib (Olumiant^®^), whose assigned targets are JAK1/2, against SARS-COV-2 infectivity, an indication for which baricitinib was recently approved^45^.

The remaining OKL hits fell into three other groups: bromodomain inhibitors (these were in OKL since bromodomains are considered atypical kinase domains) that do not bind any kinases in our analysis; selective AKT/mTOR inhibitors; and promiscuous inhibitors that share affinity for MAP4 kinases (Figure 5d-e, Supplementary Figure 4a-b). Autophagy, the tightly controlled process by which cells selectively degrade and recycle proteins and organelles, is often dysregulated in AD^46^. The finding that combinations of ULK, DAPK, mTOR, and bromodomain inhibition reduces cdsRNA-mediated neuronal death suggests a role for autophagy: autophagy is downregulated by mTOR via ULK1, a key regulator of autophagy initiation^47^ and BRD4 via transcriptional control of autophagy and lysosome related genes^48^. Inhibition of MAP4 kinases, and not JAK inhibition, has recently been shown to promote neuroprotection against paclitaxel-induced peripheral neuropathy^49^. MAP4 kinases are also the assigned targets of prosetin^50^, a drug currently in clinical trials for Amyotrophic Lateral Sclerosis (ALS). Together, these results suggest that MAP4 kinases are a node of convergence for multiple neurodegenerative conditions. This use case also illustrates the value of extending the OKL dataset in a context specific manner.

### Other applications of OKL-affinity data

The second use case is an example of chemical genetics in which small molecules were used to dissect a cellular phenotype, in this case platinum-resistant high grade serous ovarian cancer (HGSOC). Platinum-based therapies are standard of care for HGSOC and commonly elicit tumor regression that is followed by resistance and recurrence^51^. We established a cell culture model of acquired cisplatin resistance by growing SNU8 cells in the presence of increasing concentrations of cisplatin until significant (but partial) resistance was achieved (Figure 5f). Screening parental and resistant SNU8 cells with the OKL at four concentrations (10 μM, 1 μM, 100 nM, and 10 nM) yielded 22 hit compounds (Figure 5g); rescreening was performed by collecting nine-point dose response curves in the absence of cisplatin. Thirteen hits were inhibitors of EGFR-family members (including 11 assigned EGFR inhibitors), four were MEK inhibitors and two were PI3K inhibitors (Figure 5h-i, Supplementary Figure 5a). These data demonstrate a requirement for EGFR signal transduction in cisplatin resistance, fulfilling our “chemical genetics” goal. They are also consistent with previous reports in cells and animal models ^52,53^. However, EGFR inhibition has not proven to be clinically effective in the treatment of platinum-refractory HGSOC^54^, but inhibition of kinases downstream of EGFR is another possibility as is inhibition of kinases not in the EGFR pathway but inhibited by hits in our screen such as GNE-3511 (a DLK inhibitor), CHIR-98014 (a GSK3 inhibitor), and UCN-01 (a broadly active compound).

In the third use case we looked for inhibitors of non-mammalian (pathogen) kinases present in the KINOMEscan panel. We identified nine inhibitors with ^KS^Kd ≤ 100 nM for PKNB (Figure 5j), a serine threonine kinase essential for growth in *M. tuberculosis* (Mtb)^55–57^. We confirmed the potency of the top hits with 11-point Kd assays and found all had Kd < 15 nM (Figure 5k). When tested in Mtb growth-inhibition assays, pacritinib achieved half-maximal growth inhibition at 22.4 μM (95% confidence interval 19.9 μM – 25.3 μM) when butyrate was the carbon source (Figure 5l) but it was not effective under dormancy conditions^58^ (Supplementary Figure 5c) consistent with the role of PKNB in Mtb growth. Since Mtb is treated with multiple drugs, we tested pacritinib in combination with eight antibiotics that are core components in standard of care treatment cocktails; we observed substantial synergy with isoniazid, pyrazinamide, and rifampicin (Figure 5m). Pacritinib is indicated for treatment of myelofibrosis and thrombocytopenia^59^ and its Cmax value is well below the effective concentration for inhibition of Mtb growth in vitro, but development of a pacritinib-like molecule based on existing medicinal chemistry series may be possible. We also identified 14 potent inhibitors (^KS^Kd ≤ 100 nM) for the *P. falciparum* kinase PFCDPK1 and one for PFPK5 (Supplementary Figure 5b). PFPK5 is structurally similar to mammalian CDKs^60^ and is most potently bound by PHA703887 whose assigned target is CDK2. This suggests that molecules intended to target human CDKs could be repurposed to develop anti-malaria drugs.

### Recommendations on future KINOMEscans and interpreting existing data

**Figure 6:**
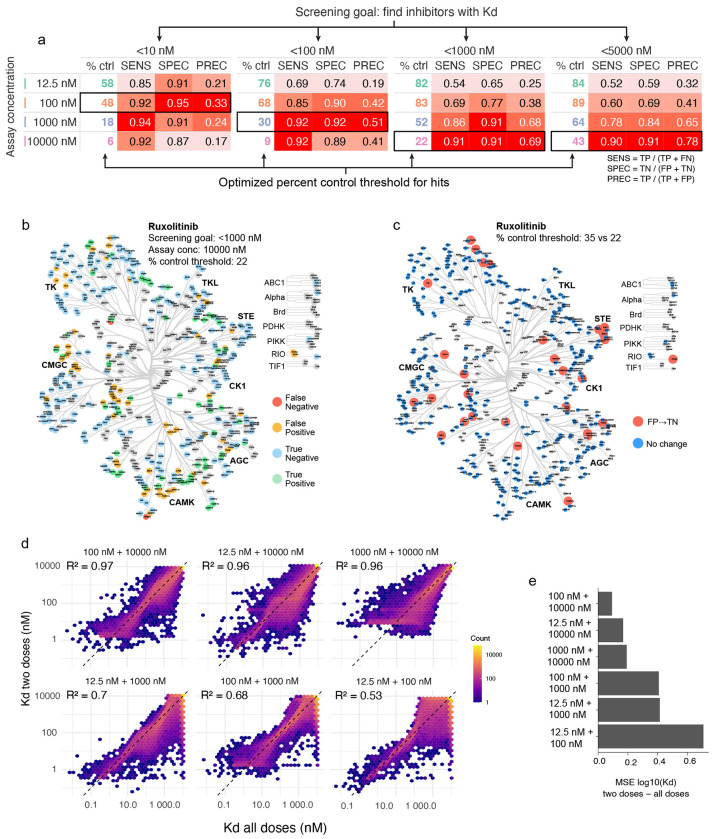
Recommendations for KINOMEscan analysis and interpretation. **(a)** Look-up table based on screening goal and assay concentration showing the sensitivity (SENS), specificity (SPEC) and precision (PREC) for the optimal cutoff values. **(b)** Kinome tree showing the classification of each kinase in a KINOMEscan of 10 μM ruxolitinib using a percent control threshold of 22. **(c)** Kinome tree showing the kinases that are misclassified using a 35% threshold and correctly classified using a 22% threshold. **(d)** Hexagonally binned scatter plots showing the ^KS^Kd values estimated from all two dose pairs with respect to those estimated from all four doses. **(e)** Summary barplot of the mean squared error (MSE) of log_10_(K_d_) for all compound–kinase pairs for ^KS^Kd values estimated from all two dose pairs.

A review of the literature suggests that it is rare to perform KINOMEscan assays at more than one dose, presumably due to cost. Eurofins suggests that hits be identified as having <35% of binding relative to a negative control regardless of the kinase or screening concentration. When we compared percent control values at each of our screening concentrations to Kds from ChEMBL (Supplementary Figure 6a) and evaluated the likelihood of correctly classifying a compound–target interaction across a range of percent control thresholds (*see* Methods, Supplementary Figure 6b–c). We found that the optimal screening concentration and threshold differed based on the objective of the screen and tolerance for false positive as opposed to false negative data (Type I and II errors; Figure 6a). For example, were one to screen ruxolitinib at 10 μM with the goal of identifying targets having Kd < 1000 nM, Eurofins standard threshold of 35% would return 94 false positives and two false negatives whereas our recommended cutoff 22% for this screening scenario returns 64 false positives and two false negatives (Figure 6b–c). This reduces false positives (by one-third) but given the amount of effort required to investigate off-targets, we also studied the value of performing KINOMEscans at two concentrations. To do this, we used Bayesian inference to estimate ^KS^Kd values when two doses were screened (see Supplementary Note, Supplementary Figure 6d, Figure 6d) and found that 10 μM and 100 nM datapoints were optimal and were well correlated with four-point data (R^2^ = 0.97, mean squared error = 0.094 for 2 dose vs. 4 dose ^KS^Kd values, Figure 6d–e). We conclude that it is likely to be more efficient to screen compounds at two doses rather than one as a means of reducing type 1 error (Figure 6e).

## DISCUSSION

The biological and clinical activities of molecules used as human therapeutics and tool compounds are commonly described in terms of a limited set of assigned targets, although it is generally understood that a substantial number of additional proteins are also bound. In some cases this can lead to striking changes in therapeutic focus and mechanism of action. Crizotinib, for example, was developed by Pfizer as an inhibitor of the cMET RTK but became a first in class inhibitor of ALK when its additional activities were discovered in cell line studies^61^ and confirmed in clinical trials^62^. It is now possible to profile kinase inhibitors against the great majority of the human kinome using assays such as KINOMEscan, but such data has only sporadically become publicly available, particularly for therapeutics. In this paper we assemble a kinase inhibitor library (OKL) that balanced competing demands of compactness, chemical diversity, target coverage, and degree of clinical development. Performing KINOMEscan assays at four doses followed by Bayesian inference of binding constants (^KS^Kd) yielded data that was well correlated with existing information in ChEMBL while filling in unknown quantities necessary for systematic analysis.

These data show that many kinase inhibitors, even those used as human therapeutics are potent but not particularly selective with a PI_max_ < 0.2, meaning that they bind multiple targets with similar affinities. Strikingly, for kinase inhibitors used as therapeutics, an assigned target is the *highest* affinity binder in only 23% of cases examined. We find no evidence that drugs that have succeeded in clinical development as opposed to those that were abandoned after Phase 1 are more selective. Moreover, the average selectivity of therapeutics has not appreciably increased over time, although the last few years have witnessed the development of some highly selective compounds. It does not follow that kinases inhibited by a particular compound are part of the biological or therapeutic mechanism of action, but it is noteworthy that these off targets include many understudied kinases whose functions are poorly understood. Thus, it is tempting to speculate that it is kinase inhibitor polypharmacology rather than selectivity that explains why this class of drugs has been so successful^63^.

The availability of systematic kinase inhibitor activity data has multiple potential uses (thus, the data in this paper are freely available in raw and processed forms and have been incorporated in smallmoleculesuite.org). One striking finding is that there exists a subset of kinases that are substantially more promiscuous than the kinome as whole. In some cases, it is possible to rationalize this in terms of known structural properties (e.g. the DFG in and out conformations) but in many others we cannot, suggesting that additional structural studies are necessary. Unrecognized patterns of selective binding are also revealing. For example, potent binding of alectinib (assigned target ALK) to the liver-specific phosphorylase kinase isoform PHKG2 may explain the observed hepatoxicity of this compound. Not all potent ALK binders also bind PHKG2, however, suggesting that it might be possible to increase therapeutic index by considering PHKG2 as an anti-target during compound development^64^. Systematic affinity data also reveal multiple cases in which closely related kinases can be differentially targeted with existing chemical backbones, as well as cases in which this is not possible; such information can guide screening and medicinal chemistry efforts. The spectrum of small molecule binding data for each kinase also represents a different way to construct a kinome tree. Currently this is based on sequence alignment and presumed phylogeny, but a tree constructed by clustering small molecule data would be substantially more useful for drug discovery and mechanism of action studies. We estimate that a roughly two-fold expansion of the OKL library to include the latest clinical and pre-clinical traditional kinase inhibitors, PROTACs and molecular glues^65^ would make this possible and is well within the capacity of an academic lab with dedicated funding. The current paper includes computational tools and practical guidelines to guide such an effort, or any other use of KINOMEscan screening, that go beyond what the vendor (Eurofins) recommends.

The possibility of instantiating the OKL in this paper using commercial resources makes it feasible to use it for screening. Moreover, in cases in which an inhibitor with multiple targets induces a desirable phenotype, an OKL screen can help to narrow down the list of functional targets even in settings in which RNAi and similar genetic approaches are challenging. For example, in the work of Petrova et al^49^. a multi-targeting kinase inhibitor, KW-2449, was found to be neuroprotective against paclitaxel-induced peripheral neuropathy and an OKL screen suggested that STE20 kinases play a critical role.^49^ In this paper we use OKL to screen for potential neuroprotective agents in a model of Alzheimer’s disease and platinum-resistant ovarian cancer. In each case, the availability of systematic binding data made the screens much easier to interpret because they revealed common and, in some cases, unrecognized off-target activities. These studies also demonstrated that it is possible to selectively expand the OKL and associated data to include new molecules focused on specific kinase subsets (in our case JAK family inhibitors).

Drugs targeting kinases are indicated for many conditions, and kinase inhibition remains an area of intense focus in academia and pharma. Despite decades of effort, moving new molecules through development and predicting which are most likely to make good therapeutics remains challenging. The current work challenges some common assumptions about what makes a good human therapeutic, while providing an approach to maximizing the information that can be obtained from phenotypic screens.

## METHODS

### KINOMEscan assays

Small molecule inhibitors were purchased from commercial vendors. Stock solutions were prepared in dDMSO at 10 mM for all compounds with the following exceptions due to limitations in solubility: CAY10561 5 mM, CHIR-98104 2 mM, gedatolisib 4 mM, and indirubin 3 mM. Inhibitors were arrayed in 96 well plates, 100 μl per inhibitor, and sent to Eurofins for scanMAX profiling. scanMAX profiling was performed in two batches: in the first batch, scans were performed at 10 μM and 100 nM; scans in the second batch were performed at 1 μM and 12.5 nM concentrations. Due to space constraints on assay plates, a subset of inhibitors was only screened in the first batch and therefore only have data points at 10 μM and 100 nM. For each concentration of each inhibitor, a percent of control value was returned for 406 WT kinases and 59 mutant kinases and three non-mammalian kinases for analysis.

Inhibitors were sent to Reaction Biology for follow-up IC50 assays, performed in duplicate at 10-dose points (HotSpot^™^ radiometric assays, with 10 μM ATP), or to Eurofins for follow-up Kd assays performed in duplicate at 11-dose points (KdELECT^®^) depending on assay availability.

### Quality control of KINOMEscan data

To assess the quality of the dataset, we first examined its internal consistency by plotting the percent control values in four-point dose response curves for every inhibitor-kinase pair and classifying their shapes. Eurofins recommends using a cutoff of 35% control for hits. We defined five classes: *non-binding*, no measurements below 35% control; *binding*, two or more measurements below 35% control, all measurements concordant; *weakly binding (high confidence)*, one measurement below 35%, p<0.1 for a linear model fit to the dose response curve; *weakly binding (low confidence)*, none of the above apply (these are usually curves where the 10 μM data point is the only data point below 35%); and *discordant*, one measurement below 35% control, one measurement, at a higher concentration, above 35% and at least two-fold higher than a previous value (Supplementary Figure 1a). We found that 73% of the dose responses measured were classified as non-binding in line with the library consisting primarily of inhibitors that were designed to be selective. However, 2% of the ~90k dose response relationships were classified as discordant, accounting for 6% of the data points when non-binding curves are excluded (Supplementary Figure 1b). When we took a closer look at the discordant class, particular inhibitors and kinases accounted for a disproportionate number of these aberrant curves suggesting that there were systematic errors in the dataset (Supplementary Figure 1c, d). The dose response relationships for these kinases and inhibitors showed clear batch effects (the percent control values for the 1 μM and 12.5 nM data points were often lower than the 10 μM and 100 nM data points) (Supplementary Figure 1e–g, left panels). Kinases are pooled in KINOMEscan assays so the worst performing kinases were likely multiplexed during data collection. Therefore, we repeated the assays for all inhibitors for the seven worst performing kinases (Supplementary Figure 1d, bold font), and for the 12.5 nM and 1000 nM concentrations of erlotinib (the worst performing inhibitor). This markedly improved the concordance of the dose responses for those conditions (Supplementary Figure 1e–g, right panels) and reduced the overall fraction of discordant dose responses to 1% of the total dataset (4% if non-binding curves are excluded) (Supplementary Figure 1h). The remaining discordant dose responses are broadly distributed across kinases and inhibitors suggesting that any remaining errors were random (Figure 2g). Discordant dose responses have been removed from all analyses. There were an additional 300 data points that were initially reported as 100% control (no binding) that were qPCR dropouts (Eurofins provided this list, but it is not routinely shared). These data points have also been excluded.

Inhibitor type assignments were sourced from the literature as much as possible. The relative affinity of kinase inhibitors for WT or mutant ABL1 in its nonphosphorylated vs. phosphorylated states has been previously shown to distinguish type I (no preference for activation state) from type II inhibitors (preference for the nonphosphorylated state)^7,66^. We used this approach for inhibitors with Kd < 3 μM for any ABL1 target.

### KINOMEscan-derived Kd values (^KD^Kd), target affinity spectra, and partition index calculations

To estimate dissociation constants (Kd) and Hill slopes from KINOMEscan dose-response data, we implemented a Bayesian hierarchical model using PyMC (version 5.25.1) with the nutpie sampler (version 0.15.2). The model relates compound concentration to the observed percent control values through a standard dose-response relationship. The mean response at a given dose was modeled using a variation of the four-parameter Hill equation:

$$\mu (c)=100-\frac{100}{1+{\left(\frac{K_{d}}{c}\right)}^{h}}$$

where *c* is the compound concentration, *K_d_* is the dissociation constant, and *h* is the Hill slope coefficient. This formulation represents the percent of kinase activity remaining (percent control) as a function of inhibitor concentration.

The measurement error was modeled as heteroscedastic, with variance proportional to the predicted response. We used an empirically derived error model:

σ(μ)=max(0.15μ−0.043,1)

Where σ is the standard deviation of the measurement error at predicted response *μ*. This error model was derived from technical reports provided by the vendor and accounts for the observation that measurement variability increases with higher percent control values, while maintaining a minimum error floor of 1% control.

We employed weakly informative priors to constrain parameters to biologically plausible ranges while allowing the data to dominate inference. $\log\left(K_{d}\right)∼𝒩\left(\log\left(10^{-6}\right),3\right))$, corresponding to a log-normal prior on *K_d_* centered at 1 μM with wide variance spanning nanomolar to millimolar affinities; and log(h)∼𝒩(0,0.5) corresponding to a log-normal prior on the Hill slope centered at 1

The observed percent control values were modeled as:

$$y_{i}∼𝒩\left(\mu \left(d_{i}\right),\sigma \left(\mu \left(d_{i}\right)\right)\right)$$

Where $y_{i}$ is the observed percent control at dose $d_{i}$

For each compound-kinase pair, we sampled from the posterior distribution using the No-U-Turn Sampler (NUTS) as implemented in the nutpie package. We ran 4 independent Markov chains, each with 1,000 tuning iterations and 2,000 sampling iterations, for a total of 8,000 posterior samples per compound-kinase pair. Convergence was assessed using the Gelman-Rubin statistic $\left(\hat{R}\right)$, with $\hat{R}<1.01$, considered indicative of convergence. Point estimates for *K_d_* and Hill slope were obtained as posterior means, with uncertainty quantified using posterior standard deviation and 95% highest density intervals.

To facilitate comparisons with previous analyses, we converted the ^KD^Kd_app_ values to target affinity spectra (TAS): ^KD^Kd_app_ > 10 μM corresponds to a TAS value of 10; ^KD^Kd_app_ between 1 μM and 10 μM to TAS 3; ^KD^Kd_app_ between 100 nM and 1 μM to TAS 2; and ^KD^Kd_app_ < 12.5 corresponds to TAS 1.

We calculated the partition index^21,67^ (PI) for each kinase and inhibitor pair using our ^KD^Kd_app_ values (equation 3).


Equation 1: PI=1/Kd(target of interest)/sum(1/Kd1…1/Kdn)(all targets).

### OKLv2 screening in ReNcell^®^ VM cells

ReN VM cells were maintained in their recommended growth conditions in ReNcell^®^ NSC maintenance media (EMD Millipore, Danvers MA), supplemented with 20 ng/ml rhEGF (Millipore Sigma, Burlington, MA), 20 ng/ml FGF (Millipore Sigma, Burlington, MA); 5% CO_2_, 37°C, on growth factor reduced Matrigel (Corning, Corning, NY). Prior to drug treatments, cells were differentiated on 10 cm plates by removing growth factors from the media for seven days. Cells were then removed from the plates using accutase (EMD Millipore, Danvers, MA) and plated in 96 well plates and allowed to differentiate for another seven days. Media was changed twice per week during differentiation. For OKLv2 screening (final concentration 1 μM), drug treatments were added by acoustic transfer using an ECHO 655 (Beckman Coulter, Brea, CA) at the ICCB-L screening facility one hour prior to the addition of 2 μg/ml (final concentration) of poly(I:C) HMW (Invivogen, San Diego, CA). After four (replicate 1) or six (replicates 2 and 3) days, an equal volume of Cell Titer Glo 2.0 (Promega, Madison, WI) was added to each well, incubated at room temperature with rocking for 10 min. and luminescence was measured with a Synergy H1 (BioTek, Winooski, VT) plate reader. Validation experiments were performed in the same way except drug treatments were delivered with a D300 digital drug dispenser, and data were collected after seven days.

### Dose response measurements in ovarian cancer cells

SNU8 ovarian cancer cells were maintained in RPMI-1640 supplemented with 2 mM L-glutamine, 25 mM HEPES, 10% fetal bovine serum and 1% penicillin/streptomycin at 37°C and 5% CO2. Cells were identity validated by short tandem repeat profiling^68^ and verified to be free from mycoplasma. Cisplatin-resistant cells were generated by adding cisplatin to the growth media starting at 0.5 μM. The dose was escalated incrementally, as the cells allowed, until they were stable in the presence of 3 μM cisplatin (approximately six months). For all dose response measurements, the parental and resistant cells were plated in 384 well CellCarrier ULTRA plates (Perkin Elmer, Waltham, MA) at 750 cells per well in 60 μl media, in the absence of cisplatin. Cells were allowed to adhere for 24 h prior to drug treatment. Drug treatments (final concentrations of 10, 1, 0.1, and 0.01 μM) were delivered via pin transfer with a custom E2C2515-UL Scara robot (Epson, Long Beach, CA) coupled to stainless steel pins (V&P Scientific, San Diego, CA) from 1000x library plates at the ICCB-L screening facility at Harvard Medical School for the screens, and using a D300 digital drug dispenser in nine-point half-log dilution series for validation experiments (Hewlett-Packard, Palo Alto, CA). At the same time that drugs were added, a time = 0 plate was stained and fixed using the Deep Dye Drop protocol^69^. Following six days in drug, all remaining assay plates were stained and fixed using the Deep Dye Drop protocol. In brief, live cells were stained with LIVE/DEAD Red (LDR) (Thermo Fisher, Waltham, MA), and pulsed with EdU (Lumiprobe, Waltham, MA) for one hour in a solution of 10% Optiprep^™^ (a density gradient medium) (Sigma, St. Louis, MO) in phosphate buffered saline (PBS). Cells were then fixed with 4% formaldehyde prepared in 20% Optiprep^™^ for 30 min. The addition of sequentially denser solutions enables staining and fixation without the need for any aspirate or wash steps thus preserving cells susceptible to loss, reducing the number of protocol steps, and sparing reagents and costs^69^. Following fixation, cells were permeabilized with 0.5% Triton X-100 for 15 min., the EdU was labeled with cy3-azide (Lumiprobe, Waltham, MA) using Click chemistry for 30 min., cells blocked in Odyssey buffer (Li-COR Biosciences, Lincoln, NE) for one hour at room temperature, and finally stained overnight with Hoechst (1:5000, Thermo Fisher, Waltham, MA) and an alexa 488 conjugated antiphospho histone H3 antibody prepared in Odyssey (1:2000, Cell Signaling Technologies, Danvers, MA).

Stained and fixed cells were imaged with an ImageXpress Micro-Confocal (IXM-C) high throughput microscope equipped with a plate hotel and robotic arm (Molecular Devices, San Jose, CA). Four fields of view were imaged per well with a 10x objective for full well coverage. Image analysis was performed with MetaXpress software (version 6.5.3.427, Molecular Devices, San Jose, CA): nuclei were segmented, and nuclear masks were defined based on the Hoechst signal. The nuclear mask was dilated to create a ring mask, and the intensity of each signal in each mask was measured. Local background was accounted-for by subtracting the intensity of each marker in the ring mask from the nuclear mask. Objects touching the edge of the images were excluded from analysis. Analysis of the raw feature data was performed using custom scripts (https://github.com/datarail/DrugResponse/wiki)^69^. The integrated Hoechst intensity was used for DNA content quantification, EdU intensity for S-phase cells, and phospho-histone H3 intensity for M-phase cells. LDR signal was used to identify dead cells. Growth rate inhibition (GR) values and metrics were calculated as defined by the number of viable cells at the time of drug treatment (t=0), and at endpoint under untreated and treated conditions^70^. A compound was considered to induce a stronger effect in the cisplatin resistant cells if the difference between the area over the GR curve (GR_AOC_) was at least 0.2 lower than for the same treatment in the parental controls. Compounds that met this condition in at least two biological replicates of the screen were considered hits.

### Molecular Dynamics Simulations

We performed molecular dynamics (MD) simulations to investigate the structural and energetic basis of CLK-inhibitor interactions for both apo and holo conformations of CLK1 (PDB 6Q8K), CLK2 (PDB 6FYI), CLK3 (PDB 6RCT), and CLK4 (PDB 6FYV) with eight inhibitors (64 simulations in total) using the GROMACS^71,72^ molecular simulation platform. We constructed two system configurations: (1) *apo-CLK systems*, where the inhibitor was positioned near CLK but outside the binding pocket, and (2) *holo-CLK systems*, where the inhibitor was positioned within the CLK binding pocket. We applied the charmm36 force field^73^ to model the systems and parameterized the inhibitors using the Automated Topology Builder (ATB)^74^. To prepare the initial system configurations, we solvated CLK-inhibitor in a cubic simulation box with the TIP3P water model and added Na^+^ or Cl^−^ ions to neutralize the charge and avoid artifacts in long-range electrostatics calculations. We carried out energy minimization using the steepest descent method^75^, followed by a 100-ps equilibration phase under the canonical ensemble (NVT) at 300 K, where we restrained the protein’s heavy atoms. We then carried out isothermal-isobaric equilibration (NPT) at 300 K and 1 bar using the Parrinello-Rahman barostat^76^. Finally, we conducted production MD simulations for 500 ns under the same thermodynamic conditions, using a time step of 2 fs. All equilibration and production runs used the leapfrog integrator^77^. We imposed periodic boundary conditions in all dimensions and calculated long-range electrostatics using the Particle Mesh Ewald (PME) method^78^. To ensure consistency, the same force field parameters and simulation protocols were applied across all systems. Predicted scores were normalized to the highest absolute value of all predictions.

### Dose response measurements in *M. tuberculosis*

Aliquots of pacritinib, AT9283, GSK690693, and GSK1070916 (200 μl at 10 mM in DMSO) were sent to the Aldridge lab at Tufts University. Dose centering curves were performed for *M. tuberculosis* (Mtb) in standard, butyrate, high cholesterol, acidic, and dormancy growth conditions^58^.

## Supplementary Material

1
