# Supplementary material for: Polypharmacology of an Optimal Kinase Library
Source: bioRxiv. 2026 Mar 19:2026.03.17.711623. Preprint. [Version 1] doi: 10.64898/2026.03.17.711623 (PMC13015435; doi:10.64898/2026.03.17.711623)
Supplement: Supplementary file 1 [file NIHPP2026.03.17.711623v1-supplement-1.pdf]

bioRxiv preprint doi: https://doi.org/10.64898/2026.03.17.711623; this version posted March 19, 2026. The copyright holder for this preprint (which was not certified by peer review) is the author/funder, who has granted bioRxiv a license to display the preprint in perpetuity. It is made available under aCC-BY 4.0 International license.

Supplementary Note

Bayesian Estimation of Dissociation Constants from four- and two-dose KINOMEscan Dose-Response Data

The Eurofins KINOMEscan platform provides a cost-effective competitive binding assay for profiling compound affinity across nearly the entire human kinome. In this study, we profiled each of the 192 OKL compounds at four concentrations (12.5, 100, 1000, and 10,000 nM), generating 89,856 four-point dose-response curves. Conventionally, KINOMEscan data are assessed at a single concentration using a threshold of <35% control remaining to classify a compound–kinase pair as a 'hit.' While straightforward, this binary classification discards quantitative information about binding affinity and provides no estimate of the dissociation constant (K_d). To extract more information from these dose-response data, we utilized a Bayesian inference framework that fits a sigmoidal dose-response model to the observed percent control values, yielding posterior distributions over K_d and Hill slope for each compound–kinase pair.

Model Specification

We model the relationship between inhibitor concentration and the observed percent of control remaining using a four-parameter Hill equation. Let c denote the compound concentration, K_d the dissociation constant, and h the Hill slope coefficient. The mean predicted response is:

$$\mu(c) = 100 - 100 / (1 + (K_d / c)^h)$$

This formulation represents the percent of kinase activity remaining as a function of inhibitor concentration: at low concentrations relative to K_d, the predicted response approaches 100% (no inhibition), and at high concentrations it approaches 0% (complete inhibition).

Measurement error was modeled as heteroscedastic, with the standard deviation proportional to the predicted response. We employed an empirically derived error model based on technical reports from Eurofins:

$$\sigma(\mu) = \max(0.15\mu - 0.043, 1)$$

This captures the observation that measurement variability increases with higher percent control values while maintaining a minimum error floor of 1% control. The observed percent control values y_i at each dose d_i were modeled as normally distributed around the predicted mean with standard deviation given by the error model.

Prior Distributions

We specified weakly informative priors to constrain parameters to biologically plausible ranges while allowing the data to dominate inference. For the dissociation constant, we placed a log-normal prior centered at 1 μM with wide variance:

$$\log(K_d) \sim N(\log(10^{-6}), 3)$$

This prior spans a broad range from sub-nanomolar to millimolar affinities, accommodating the full spectrum of biologically relevant dissociation constants. For the Hill slope, we used:

$$\log(h) \sim N(0, 0.5)$$

bioRxiv preprint doi: https://doi.org/10.64898/2026.03.17.711623; this version posted March 19, 2026. The copyright holder for this preprint (which was not certified by peer review) is the author/funder, who has granted bioRxiv a license to display the preprint in perpetuity. It is made available under aCC-BY 4.0 International license.

corresponding to a log-normal prior centered at a Hill slope of 1, consistent with standard single-site binding kinetics, while allowing moderate deviations.

Posterior Inference

For each compound–kinase pair, we sampled from the posterior distribution using the No-U-Turn Sampler (NUTS) as implemented in the nutpie package with PyMC (version 5.25.1). We ran four independent Markov chains, each with 1,000 tuning iterations and 2,000 sampling iterations, yielding 8,000 posterior samples per pair. Convergence was assessed using the Gelman–Rubin statistic ($\hat{R}$), with $\hat{R} < 1.01$ considered indicative of adequate convergence. Point estimates for K_d and Hill slope were obtained as posterior medians, and uncertainty was quantified using posterior standard deviations and 95% highest density intervals (HDIs). The full implementation is available as a Python command-line tool that supports parallel execution across compound–kinase pairs with results stored in a SQLite database for reproducibility and seamless continuation after interrupted runs.

Validation Against ChEMBL Reference Data

To assess the accuracy of our Bayesian K_d estimates (denoted $^{KS}K_d$), we compared them to K_d values deposited in ChEMBL for overlapping compound–kinase pairs where full dose-response curves had been collected using orthogonal assay platforms. We observed good agreement between the four-dose KINOMEscan-derived K_d values and the ChEMBL reference values ($R^2 = 0.91$; Figure 1f), demonstrating that four concentration points spanning four orders of magnitude are sufficient to estimate dissociation constants with high fidelity using the Bayesian framework.

Minimum Number of Doses Required for Reliable K_d Estimation

Given the cost of kinome-wide profiling, we investigated whether fewer than four concentration points could yield reliable K_d estimates. We systematically evaluated all six pairwise combinations of the four assay concentrations (12.5, 100, 1000, and 10,000 nM) by fitting the Bayesian model to each two-dose subset and comparing the resulting K_d estimates to those obtained from the full four-dose data (**Figure 6a**).

As an illustrative example, **Supplementary Figure 6a** shows dose-response fits for lenvatinib binding EPHB6 across all dose combinations. The four-dose fit (all doses) yields a K_d of 35 nM with a narrow 95% HDI. Two-dose subsets that include concentrations spanning the widest dynamic range—particularly 100 nM + 10,000 nM—produce K_d estimates (41 nM) and credible intervals closely matching the four-dose fit. In contrast, subsets comprising closely spaced concentrations (e.g., 12.5 nM + 100 nM) yield wider credible intervals and slightly different point estimates, reflecting the reduced information content of narrowly spaced doses.

To quantify performance across the entire dataset, we compared the two-dose and four-dose Kd estimates using hexagonally binned scatter plots and computed the R^2, showing that certain assay concentration combinations perform better for different ranges of Kd estimation (**Figure 6a**). To quantify the estimation error when going from four to two doses, we computed mean squared error (MSE) of $\log_{10}(K_d)$ for all compound–kinase pairs (**Figure 6b**). The combination of 100 nM + 10,000 nM achieved the best overall performance, with $R^2 = 0.97$ and MSE = 0.094. This was closely followed by 12.5 nM + 10,000 nM ($R^2 = 0.96$, MSE = 0.168) and 1000 nM + 10,000 nM ($R^2 = 0.96$, MSE = 0.193) (**Figure 6a-b**. Dose pairs that did not include the highest concentration (10,000 nM) performed substantially worse, with MSE values 2–7-fold higher.

The strong performance of the 100 nM + 10,000 nM combination can be understood intuitively: these two concentrations bracket the K_d values of most compound–kinase pairs in the dataset,

bioRxiv preprint doi: https://doi.org/10.64898/2026.03.17.711623; this version posted March 19, 2026. The copyright holder for this preprint (which was not certified by peer review) is the author/funder, who has granted bioRxiv a license to display the preprint in perpetuity. It is made available under aCC-BY 4.0 International license.

providing information about both the upper and lower plateaus of the dose-response curve. Concentrations that are both above or both below the K_d provide redundant information about one plateau, yielding poorly constrained estimates. Based on these results, we recommend that future KINOMEscan experiments adopt a minimum two-dose design at 100 nM and 10,000 nM when full four-dose profiling is not feasible due to cost constraints.

Advantages of the Bayesian Approach

For comparison to an alternative approach, we calculated Kd values for each concordant dose response that crossed 50% control by interpolating from a linear relationship between the data points on either side of the 50% mark. We assigned curves that never reached 50% control Kd > 10 μM (highest screening dose), and those that were below 50% control at all screening concentrations Kd < 100 nM or Kd < 12.5 nM depending on the lowest dose screened for each inhibitor. While the correlation between these Kd estimates and data in ChEMBL was good (R=0.88), we could not estimate relative affinities below our lowest screening concentration (**Supplementary Figure 1h**). Answers to the common questions of (i) what is the highest affinity target of an inhibitor? and (ii) what target is most selectively bound by an inhibitor? were difficult to answer since they were multi-way ties. For example, a potent multi-targeting inhibitor like dasatinib, inhibits 48 kinases with Kd < 12.5 nM, resulting in a 48-way tie for PI_{max}. With the Bayesian approach described here, we can see that dasatinib most selectively inhibits EPHA2 with PI_{max} = 0.32 and ^{KS}Kd = 0.035 nM.

The Bayesian framework described here offers several advantages over conventional threshold-based analysis of KINOMEscan data. Most fundamentally, each K_d estimate is accompanied by a full posterior distribution, from which 95% highest density credible intervals are derived. This enables downstream analyses to propagate uncertainty rather than relying on point estimates alone, providing a principled measure of confidence for each compound–kinase interaction. The weakly informative priors further serve as a form of regularization, shrinking K_d estimates derived from noisy or sparse data toward biologically plausible values. This is particularly important for compound–kinase pairs near the limit of detection, where conventional curve-fitting approaches may yield unstable or biologically implausible estimates.

Because the model encodes the expected shape of the dose-response relationship through the Hill equation and prior distributions, it can also estimate K_d values that fall outside the range of tested concentrations. Compounds with K_d < 12.5 nM (below the lowest tested concentration) or K_d > 10,000 nM (above the highest) can still be assigned meaningful, albeit less certain, affinity estimates, with broader credible intervals that appropriately reflect the increased uncertainty. This capacity for extrapolation has practical consequences for selectivity analysis: quantitative K_d estimates across the full affinity range enable the computation of continuous selectivity metrics such as the partition index (PI), which represents the fraction of compound bound to each kinase in a theoretical equimolar mixture. By leveraging the full range of estimated affinities, including sub-nanomolar K_d values extrapolated below the lowest assay concentration, the partition index substantially improves the resolution of selectivity profiles relative to threshold-based hit calling, which treats all sub-threshold interactions as equivalent.

Finally, as demonstrated above, the Bayesian framework yields reliable K_d estimates from as few as two concentration points, enabling significant cost reductions without substantially compromising data quality. This makes kinome-wide profiling more accessible and scalable for large compound collections. Notably, numerous past KINOMEscan experiments have already been performed at two doses; these existing datasets could benefit from reanalysis using the approach described here.

bioRxiv preprint doi: https://doi.org/10.64898/2026.03.17.711623; this version posted March 19, 2026. The copyright holder for this preprint (which was not certified by peer review) is the author/funder, who has granted bioRxiv a license to display the preprint in perpetuity. It is made available under aCC-BY 4.0 International license.

SUPPLEMENTARY FIGURES

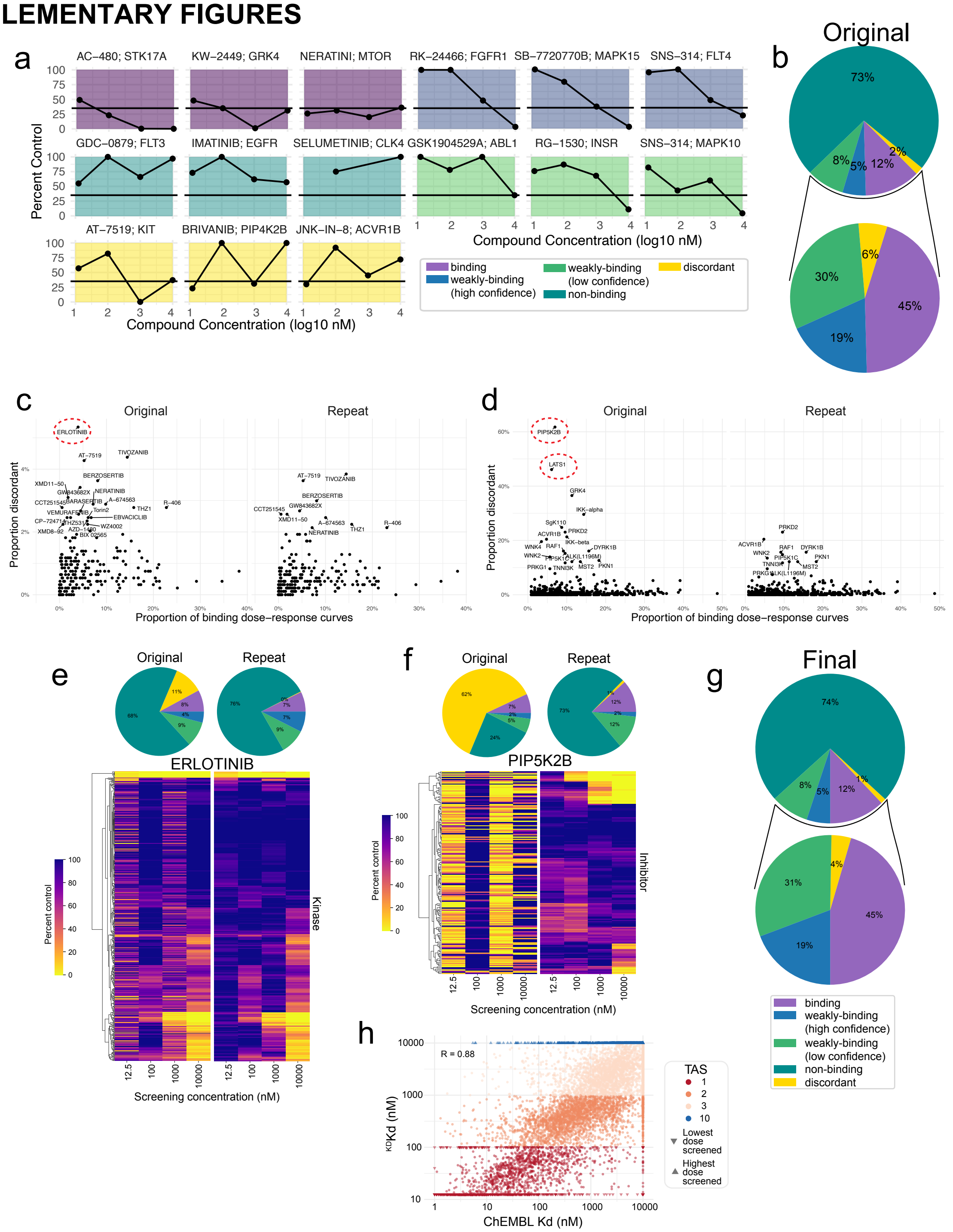

Supplementary Figure 1: **(a)** Examples of percent control dose response curves for each class as indicated. Discordant dose responses were excluded from the dataset. **(b)** Pie charts showing the fraction of dose responses that fall into each class, and the distribution of dose response classes when the confirmed non-binding class is excluded. **(c)** Scatter plots showing the fraction of discordant dose response curves with respect to the fraction of binding dose response curves for all inhibitors and **(d)** for all kinases in the original dataset (left plots) and in the final post-QC dataset (right plots). **(e)** Heatmaps showing % control values for erlotinib against all kinases at all concentrations tested, and pie charts summarizing the fraction of dose responses in each class pre- and post-QC. **(f)** Heatmaps showing % control values for PIP5K2B and **(g)** Pie charts showing the fraction of dose responses that fall into each class, and the distribution of dose response classes when the confirmed non-binding class is excluded. **(h)** Scatterplot comparing ^{KS}Kd values estimated by linear interpolation to those previously available in ChEMBL. Data points are colored by TAS value, and the values for each compound's assigned target(s) are outlined in black.

bioRxiv preprint doi: https://doi.org/10.64898/2026.03.17.711623; this version posted March 19, 2026. The copyright holder for this preprint (which was not certified by peer review) is the author/funder, who has granted bioRxiv a license to display the preprint in perpetuity. It is made available under aCC-BY 4.0 International license.

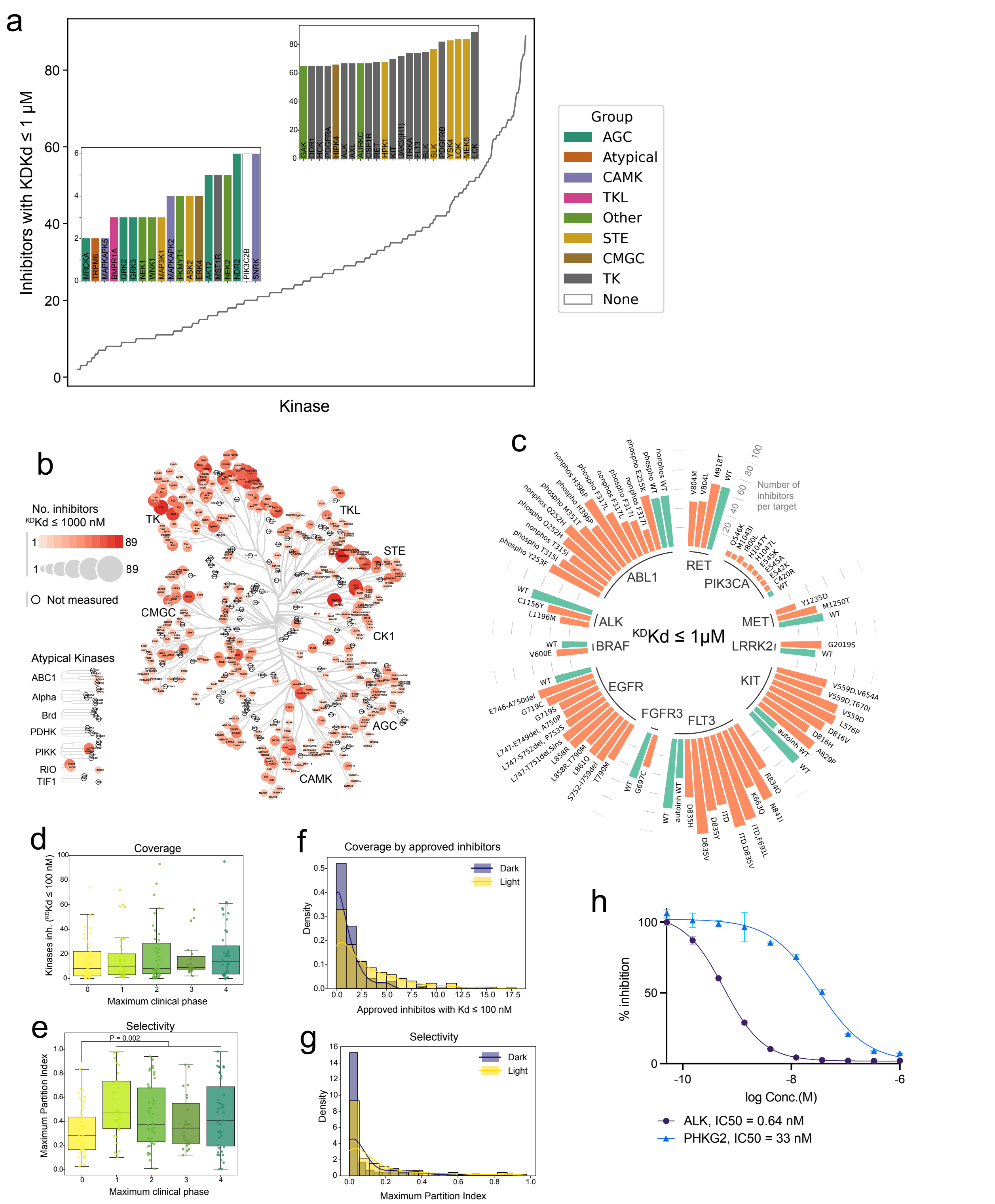

Supplementary Figure 2: **(a)** The number of inhibitors with ^{KS}Kd ≤ 1 µM per kinase. The inset plot shows the top 20 kinases most widely inhibited by OKL compounds colored by group. **(b)** The number of OKL inhibitors (^{KS}Kd < 1 µM) per kinase shown on the kinome tree by the size and intensity of the markers. **(c)** Radial bar plot showing the number of OKL inhibitors with ^{KS}Kd ≤ 1 µM for all mutant kinases (red bars) assayed in the KINOMEscan panel. WT kinases are indicated with blue bars. **(d)** Boxplot showing the number of kinases per inhibitor with ^{KS}Kd ≤ 100 nM by maximum stage of clinical development reached. **(e)** Boxplot showing the maximum partition index for each OKL inhibitor by maximum stage of clinical development reached. P-value is from a one-way ANOVA with Tukey's correction for multiple comparisons. **(f)** Histogram of the maximum partition indices for all dark and light kinases. KDE lines are overlaid for visualization only.

bioRxiv preprint doi: https://doi.org/10.64898/2026.03.17.711623; this version posted March 19, 2026. The copyright holder for this preprint (which was not certified by peer review) is the author/funder, who has granted bioRxiv a license to display the preprint in perpetuity. It is made available under aCC-BY 4.0 International license.

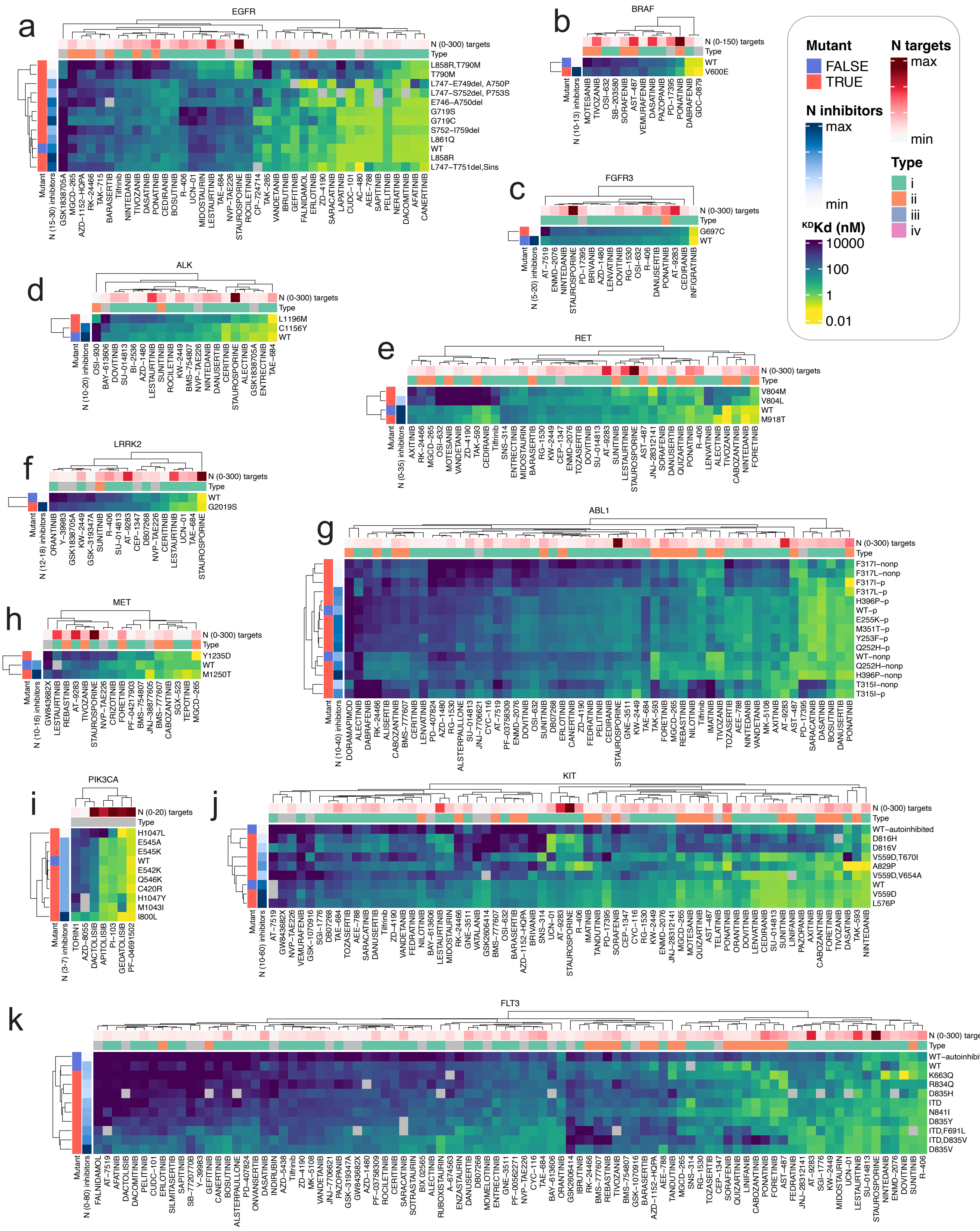

Supplementary Figure 3: **(a)** Heatmap with hierarchical clustering showing ^{KS}Kd values for OKL inhibitors against all variants of EGFR, **(b)** BRAF, **(c)** FGFR3, **(d)** ALK, **(e)** RET, **(f)** LRRK2, **(g)** ABL, **(h)** MET, **(i)** PIK3CA, **(j)** KIT, and **(k)** FLT3 that were assayed.

bioRxiv preprint doi: https://doi.org/10.64898/2026.03.17.711623; this version posted March 19, 2026. The copyright holder for this preprint (which was not certified by peer review) is the author/funder, who has granted bioRxiv a license to display the preprint in perpetuity. It is made available under aCC-BY 4.0 International license.

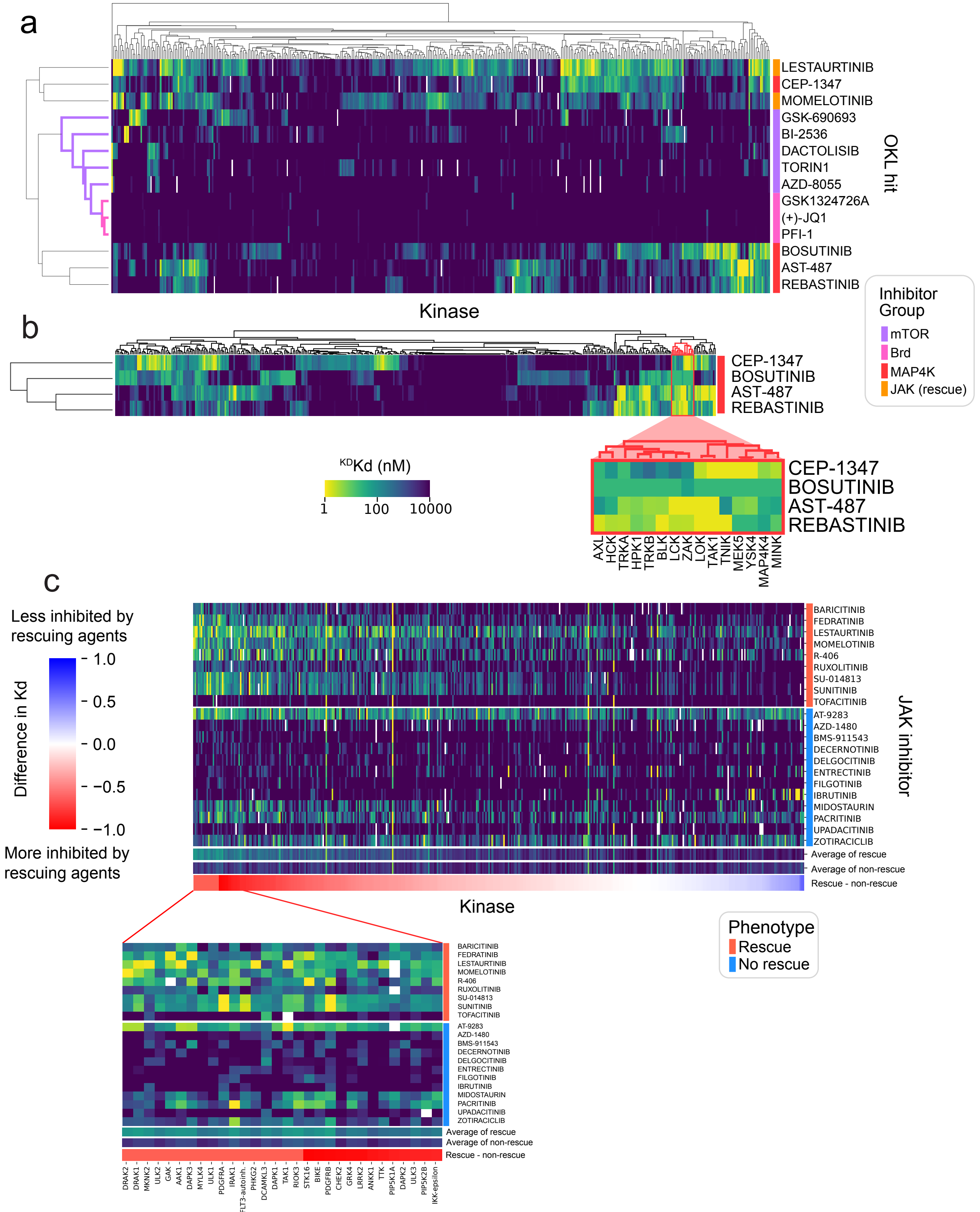

Supplementary Figure 4: **(a)** Clustermap of ^{KS}Kd values for OKL compounds that protect ReN VM cells from polyIC. The colored bars represent groupings by targets inhibited. **(b)** Clustermap of ^{KS}Kd values for the subset of OKL inhibitors denoted with a red bar in **(a)**, and a magnified view of the targets most potently inhibited by those hits. **(c)** Heatmap of ^{KS}Kd values for nominal JAK family inhibitors. Rows are sorted by phenotype; columns are sorted by the difference in ^{KS}Kd values between the rescuing and non-rescuing agents. A magnified view of the kinases that are preferentially inhibited by the rescuing agents is provided.

bioRxiv preprint doi: https://doi.org/10.64898/2026.03.17.711623; this version posted March 19, 2026. The copyright holder for this preprint (which was not certified by peer review) is the author/funder, who has granted bioRxiv a license to display the preprint in perpetuity. It is made available under aCC-BY 4.0 International license.

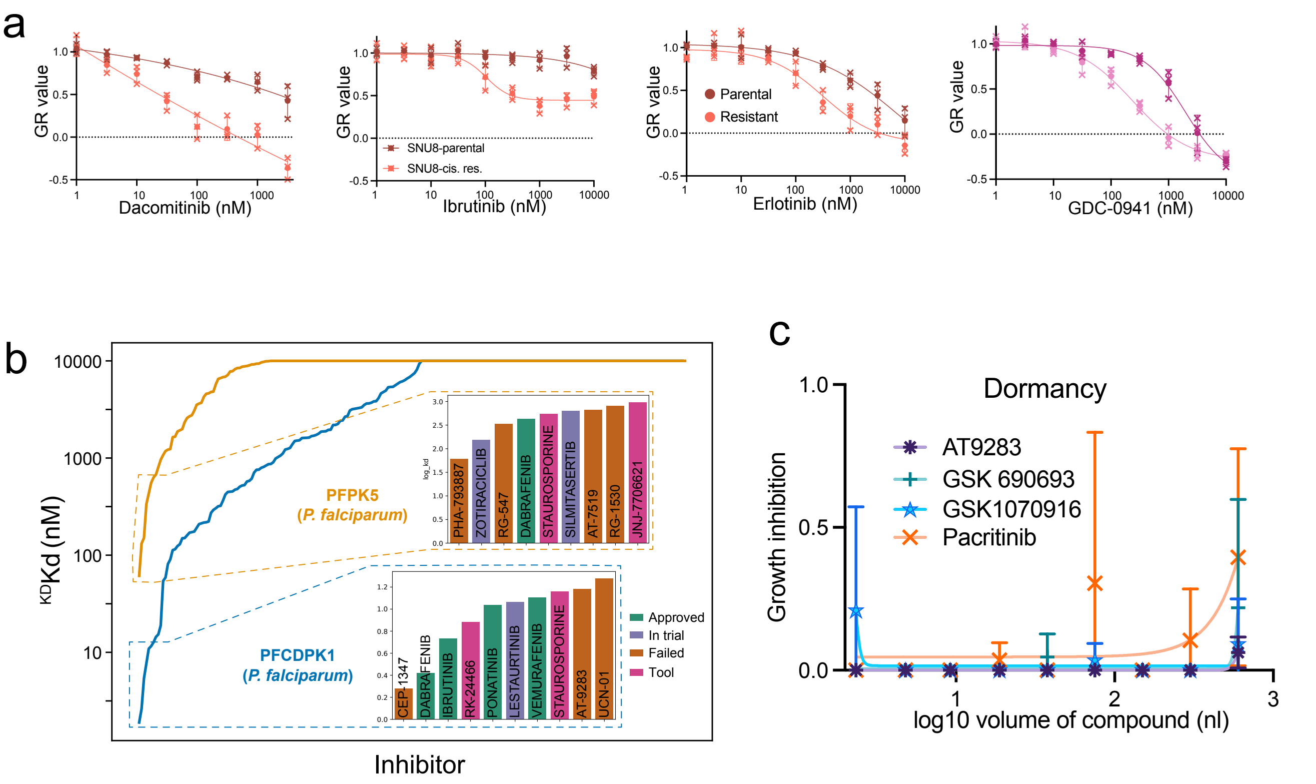

Supplementary Figure 5: **(a)** Dose response curves for the inhibitors shown in parental and cisplatin-resistant SNU8 cells after six-day treatments. **(b)** ^{KS}Kd values for OKL compounds against the *P. falciparum* (malaria) kinases, PFPK5 (orange) and PFCDPK1 (blue), the strongest inhibitors of each are shown in the inset bar graphs. **(c)** Dose-dependent growth inhibition of Mtb treated with the indicated drugs in the dormancy growth condition.

bioRxiv preprint doi: https://doi.org/10.64898/2026.03.17.711623; this version posted March 19, 2026. The copyright holder for this preprint (which was not certified by peer review) is the author/funder, who has granted bioRxiv a license to display the preprint in perpetuity. It is made available under aCC-BY 4.0 International license.

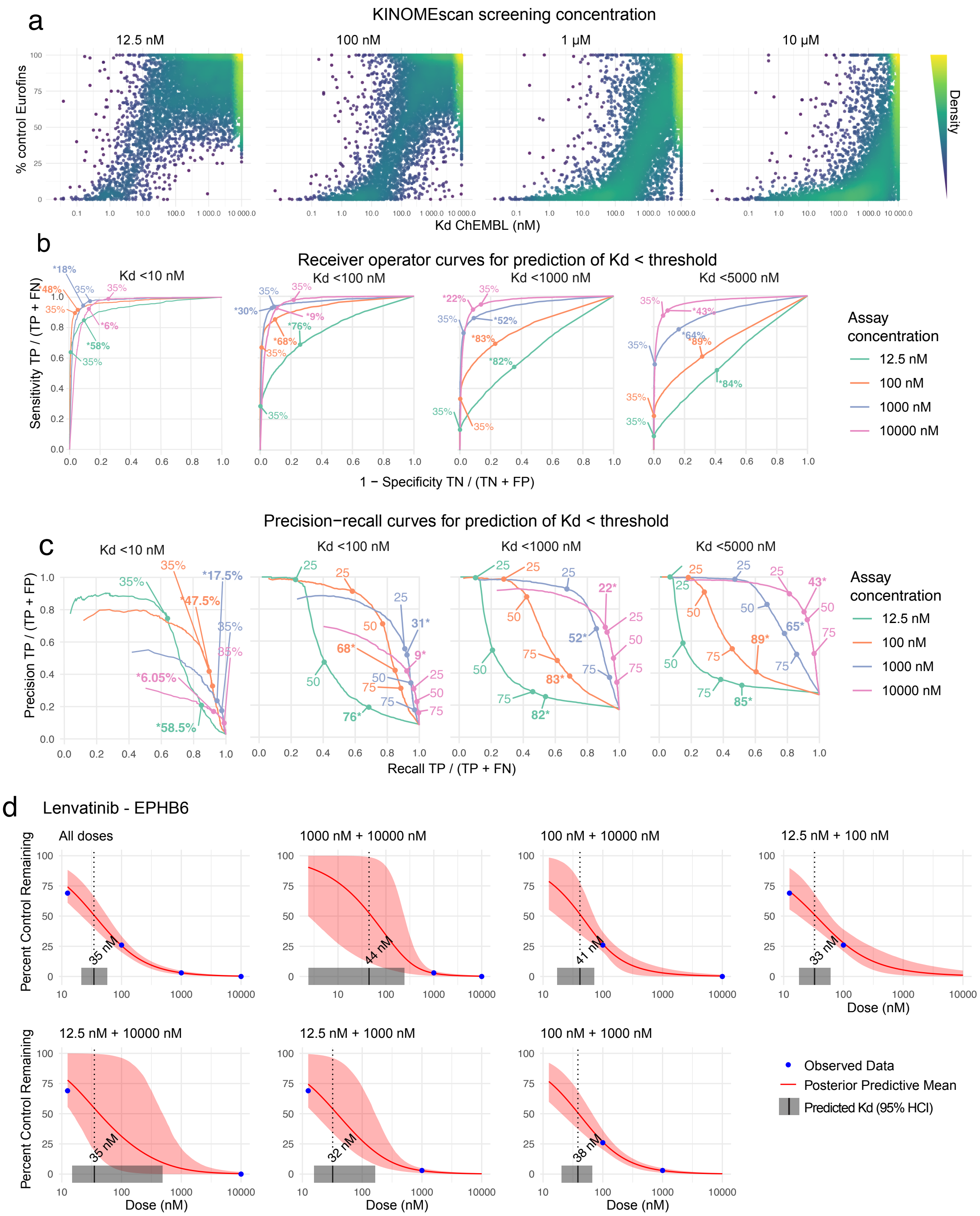

Supplementary Figure 6: (a) Scatterplots showing % control values for the screening concentrations indicated with respect to Kd data in ChEMBL. **(b)** False positive and negative rates for all percent control thresholds are plotted (taking ChEMBL data as the ground truth) on receiver operator curves, and the % control corresponding to the point closest to the upper left corner of the plot is identified as the optimal threshold. The optimal % control thresholds (denoted by * and bold font) and 35% are shown for each curve. **(c)** Precision-recall curves for predicting the Kd values shown using screening data collected at each assay concentration. The optimal % control thresholds (denoted by * and bold font) and 35% are shown for each curve. **(d)** Dose-response fits from the Bayesian model for lenvatinib binding EPHB6 across all four doses (first plot) and all two dose combinations. The credible 95% interval for the fit is shaded in red and for the ^{KS}Kd estimate in grey on the x-axis.
